# Microbiome engineering: unlocking therapeutic potential in inflammatory bowel disease

**DOI:** 10.3389/fmicb.2025.1610029

**Published:** 2025-09-23

**Authors:** Chen Kong, Long-bin Huang, Mei-feng Yang, Ning-ning Yue, Duo Luo, Yuan Zhang, Cheng-mei Tian, Yang Song, Dao-ru Wei, Rui-yue Shi, Yu-jie Liang, Jun Yao, Li-sheng Wang, De-feng Li

**Affiliations:** ^1^Department of Gastroenterology, Shenzhen People's Hospital, the Second Clinical Medical College, Jinan University, Shenzhen, Guangdong, China; ^2^Department of General Medicine, Yantian District People's Hospital, Shenzhen, Guangdong, China; ^3^Department of Geriatrics, Guangzhou First People’s Hospital, Guangzhou Medical University, Guangzhou, Guangdong, China; ^4^Department of Medical Administration, Huizhou Institute of Occupational Diseases Control and Prevention, Huizhou, Guangdong, China; ^5^Department of Emergency, Shenzhen People's Hospital (the Second Clinical Medical College, Jinan University, the First Affiliated Hospital, Southern University of Science and Technology), Shenzhen, Guangdong, China; ^6^Department of Gastroenterology, Shenzhen People's Hospital (the Second Clinical Medical College, Jinan University, the First Affiliated Hospital, Southern University of Science and Technology), Shenzhen, Guangdong, China; ^7^Department of Rehabilitation, Shenzhen People's Hospital (the Second Clinical Medical College, Jinan University, the First Affiliated Hospital, Southern University of Science and Technology), Shenzhen, Guangdong, China; ^8^Department of Child and Adolescent Psychiatry, Shenzhen Kangning Hospital, Shenzhen Mental Health Center, Shenzhen, Guangdong, China

**Keywords:** microbiome, synthetic biology, inflammatory bowel disease, extracellular vesicles, therapy

## Abstract

The human gut microbiome, traditionally linked to infectious diseases, is now recognized as a hub of non-pathogenic microorganisms that play pivotal roles in host communication and homeostasis. Advances in microbiome engineering have enabled the design of “smart” living therapeutics for inflammatory bowel disease (IBD), leveraging engineered symbiotic bacteria, yeasts, and bacteriophages. This review synthesizes recent progress in reprogramming microbes using synthetic biology tools, emphasizing their capacity to sense pathological signals and deliver targeted therapies. We critically evaluate three key approaches: synthetic gene circuits in bacteria for precision drug delivery, phage-mediated modulation of dysbiotic microbiota, and yeast-based systems for metabolic intervention (e.g., butyrate production). Challenges in biocontainment, genetic stability, and clinical translation are discussed, alongside emerging strategies such as outer membrane vesicles (OMVs) for immunomodulation. By distilling these advances, we highlight a roadmap for translating engineered microbes into safe and effective IBD therapies.

## Graphical abstract

**Figure:**
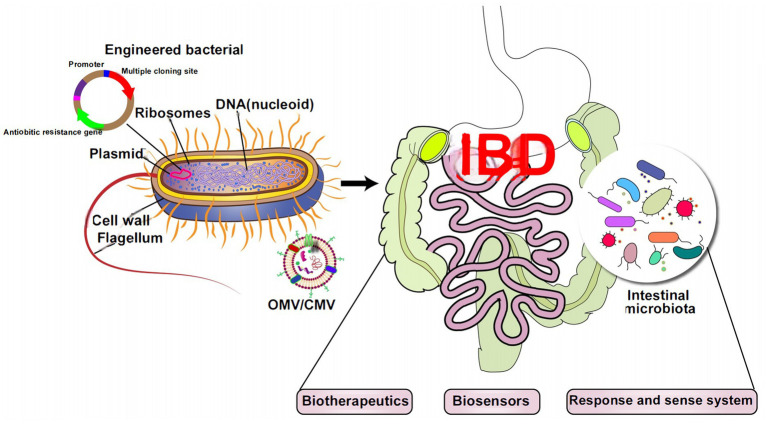
The microbiome engineering therapies in the field of IBD.

## 1 Introduction

Inflammatory bowel disease (IBD), encompassing Crohn’s disease and ulcerative colitis, presents a significant and growing global health challenge characterized by chronic intestinal inflammation. Current treatment strategies often face limitations, including variable efficacy, systemic side effects, and the potential for loss of response over time, highlighting the urgent need for novel, targeted therapeutic approaches (Riglar and Silver, 2018; Leventhal et al., 2020; Zaiss et al., 2021; Mager et al., 2020). The gut microbiome plays a crucial role in IBD pathogenesis and is increasingly recognized as a promising therapeutic target. While historically implicated in disease, recent advances in microbiome research and genomic technologies have revealed its potential for therapeutic manipulation (Charbonneau et al., 2020; Kurtz et al., 2019; Sanna et al., 2019). Microbiome engineering, particularly utilizing engineered bacteria, has emerged as a strategy with distinct potential advantages for IBD management, offering possibilities for localized diagnosis and treatment (Riglar and Silver, 2018; Leventhal et al., 2020; Zaiss et al., 2021; Mager et al., 2020). The continuous evolution of gene editing tools and synthetic biology further enables the design of bacteria with increasingly sophisticated functions, making this approach more feasible and cost-effective (Table 1; Leventhal et al., 2020; Charbonneau et al., 2020; Kurtz et al., 2019; Federici et al., 2022).

**Table 1:** Genetic engineered bacterial strains.

Target disease	Chassis	Mechanism of action	Reference
Low-grade intestinal inflammation	*Bifidobacterium bifidum*	The *Bifidobacteria* expression System (BEST) system enables *Bifidobacterium bifidum* to produce heterologous proteins (IL-10, etc.)using a broad host range plasmid, stress-inducible promoter, and two different signal peptides from *Lactococcus lactis* and *Bifidobacterium longum*.	Wright et al. (2019)
CD	*Commensal Escherichia coli*	Engineered commensal *E. coli* produce and release a biotherapeutic in response to nitric oxide (NO), a biomarker for Crohn’s disease (CD), by co-expressing transmembrane protein TolA (TolAIII) and granulocyte-macrophage colony-stimulating factor (GM-CSF).	Shuwen and Kefeng (2022)
IBD	*Escherichia coli Nissle 1917 (EcN)*	EcN produces fibrous matrices composed of curli nanofibers displaying trefoil factors (TFFs), which promote gut epithelial integrity, immunomodulation and mucosal healing.	Gogokhia et al. (2019)
UC	*Dairy Lactococcus lactis NZ9000*	Engineering of *Lactococcus lactis NZ9000* to express murine interleukin-35 (IL-35; NZ9000/IL-35) results in the accumulation of IL-35 in the gut, leading to a decrease in Th17 cells and an increase in Treg cells in the lamina propria, as well as elevated levels of IL-10 and reduced levels of pro-inflammatory cytokines IL-6, IL-17A, IFN-γ, and TNF-α in both colon tissue and serum.	Nikolich and Filippov (2020)
Crohn’s disease	*Engineered Lactobacillus casei BL23 strains*	Decreasing the levels of reactive oxygen species by producing antioxidant enzymes such as catalase (CAT) or superoxide dismutase (SOD).	Pires et al. (2021)
IBD	*Food-grade lactic acid bacteria (LAB)*	Expressing and delivering Elafin, reducing elastase activity and inflammation, preventing increased intestinal permeability, and inhibiting the release of cytokines and chemokines.	Riglar et al. (2017)
IBD	*Lactobacillus casei BL23* *Bifidobacterium*	Express manganese superoxide dismutase (MnSOD) to reduce oxidative stress and inflammation in the gut.	Chang et al. (2017) Majewska et al. (2019)
IBD	*Lactococcus lactis*	The engineered bacterium secretes the cytokine IL-10 for localized delivery.	Vandenbroucke et al. (2004)
UC	*Lactococcus lactis*	Oral administration of SlpA-expressing *L. lactis* induces higher expression of IL-27 by myeloid cells and increases IL-10 and cMAF expression in T cells.	Hsu et al. (2020)
IBD	*Lb. casei BL23*	Expression of MnKat from *L. plantarum* boosts *Lb. casei BL23* survival under oxidative stress, while sodA gene from *L. lactis* enhances MnSOD activity, reducing oxidative stress and inflammation in cell and murine colitis models.	Chang et al. (2017)
IBD	*Recombinant lactic acid bacteria (LAB)*	An IL-10 expression system regulated by stress: Stress-Inducible Controlled Expression (SICE) system.	Mimee and Nagler (2021)
IBD	*Saccharomyces boulardii*	Engineered *Saccharomyces boulardii* probiotics deliver anti-inflammatory proteins like IL-10, TNFR1-ECD, alkaline phosphatase, and atrial natriuretic peptide (ANP) locally to the gut, easing dextran sulfate sodium salt (DSS)-induced colitis in mice when orally administered.	Scott et al. (2021)
IBD	*Clostridium butyricum*	Production of butyrate and modulation of molecular and immunological signals in the digestive system, extending to other organs such as the liver, adipose tissue, and brain.	Kong et al. (2024)
IBD	*Clostridium butyricum*	Secretory overexpression of pEGF in *C. butyricum* could enhance intestinal protective functions, partly through STAT3 signal activation in IPECs	Wu et al. (2024)
UC	*Lactobacillus paracasei F19*	*Lactobacillus paracasei F19* express palmitoylethanolamide (PEA) in response to ultra-low palmitate supply, which alleviate UC symptoms.	Federici et al. (2023)
IBD	*Escherichia coli*	Bacterial strains are engineered with trigger circuits to detect specific biomarkers, such as tetrathionate, a transient product of reactive oxygen species produced during inflammation.	Zhan et al. (2019)

IBDs, Inflammatory bowel diseases; IL-10, interleukin-10; NO, nitric oxide; CD, Crohn’s disease; TolAIII, transmembrane protein TolA; GM-CSF, granulocyte-macrophage colony-stimulating factor; TFFs, trefoil factors; IL-35, interleukin-35; IFN-γ, interferon-γ; TNF-α, tumor necrosis factor-α; CAT, catalase; SOD, superoxide dismutase; MnSOD, manganese superoxide dismutase; SICE, Stress-Inducible Controlled Expression; ANP, atrial natriuretic peptide; DSS, easing dextran sulfate sodium salt; EGF, epidermal growth factor; PEA, palmitoylethanolamide; UC, ulcerative colitis.

Genetically engineered bacteria therapy offers several compelling benefits for IBD. Engineered bacteria can localize to specific sites of inflammation within the gut, areas often difficult to reach effectively with conventional systemic drugs. This targeted approach allows for direct interaction with the diseased tissue, potentially lowering off-target effects and improving safety compared to traditional administration routes (Riglar and Silver, 2018; Steidler et al., 1998; Saltzman et al., 1996). It also minimizes drug loss during systemic circulation or gastrointestinal transit, enhancing local bioavailability (Forkus et al., 2017; Hanson et al., 2014; Steidler et al., 2000; Motta, 2012; Vandenbroucke et al., 2004). As living therapeutics, engineered bacteria can be designed to sense and respond to dynamic physiological and pathological signals within the gut environment (Riglar and Silver, 2018; Riglar et al., 2017; Daeffler, 2017). This sensing ability holds promise for real-time monitoring of disease activity and drug response, providing more intuitive insights. Engineered bacteria can be programmed to interact with the host immune system, for example, by expressing immunomodulatory molecules or presenting specific antigens, thereby potentially enhancing therapeutic immune responses (Zhan et al., 2019; Sterner and Sterner, 2021).

**Figure 1:**
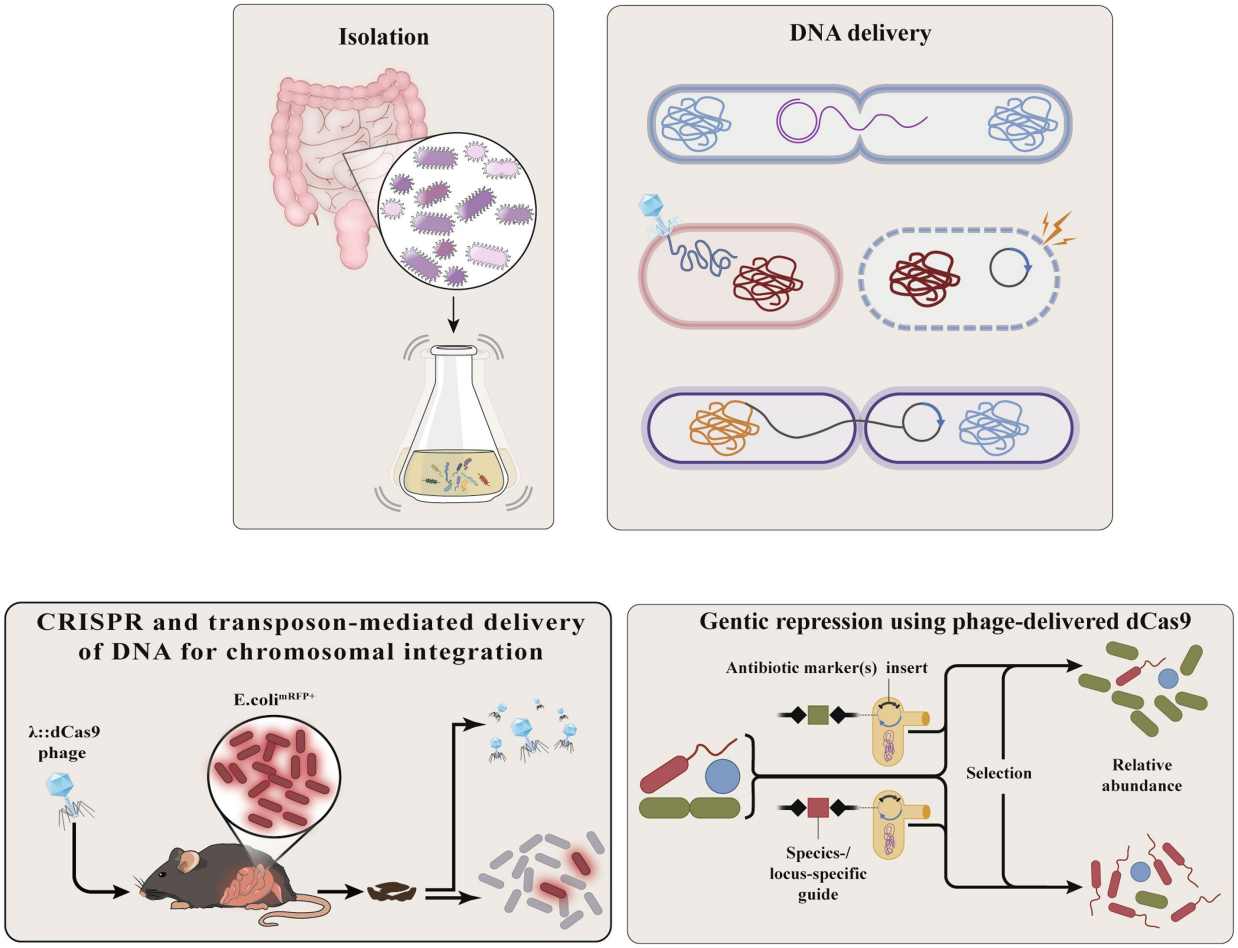
Microbiome engineering is mainly used to change the composition of microbiota or the composition or activity of active microbiota.

Given these capabilities, microbiome engineering is positioned as an emerging vehicle to diagnose and treat diseases (Riglar and Silver, 2018; Figure 1). Despite this significant promise, translating engineered microbiome therapies into clinical practice for IBD faces substantial hurdles. Key challenges include ensuring the safety and long-term stability of genetically modified organisms within the complex gut ecosystem, addressing ethical and regulatory concerns, and demonstrating consistent efficacy and viability of the engineered microbes in human patients (Riglar and Silver, 2018; Marsh and Ley, 2022). Furthermore, a comprehensive review synthesizing the latest advancements in synthetic biology tools for microbiome engineering, the design principles for therapeutic bacterial strains, the strategies for targeted delivery in the gut, and the use of novel carriers (such as bacteriophages, engineered yeast, and OMVs) specifically within the context of IBD treatment is currently lacking. This gap in the literature motivates our review.

Therefore, this review specifically focuses on the application and challenges of engineered microbiome therapeutics for IBD. We aim to discuss current developments in synthetic biology tools applied to re-program microbes into human therapeutic agents, introduce the design of engineered therapeutic strains, and evaluate practical approaches for targeted therapeutic delivery within the gastrointestinal tract. Furthermore, we elaborate on common carriers in the synthetic biology area, such as bacteriophages, engineered yeast and engineered bacteria outer membrane nanovesicles (OMVs). Finally, we discuss the perspective, future developments, and outstanding challenges of engineered microbiome therapy.

## 2 Methodology

### 2.1 Literature search strategy

This narrative review employed a structured literature search in PubMed to synthesize recent advances in microbiome engineering for IBD therapy. The search strategy combined core conceptual keywords and Medical Subject Headings (MeSH) terms:

- Primary Concepts: (“microbiome engineering” OR “synthetic biology”) AND (“inflammatory bowel disease” OR “IBD” OR “Crohn’s disease” OR “ulcerative colitis”)
- Intervention-Specific Terms:

o Engineered bacteria: (“engineered bacteria” OR “genetically modified bacteria” OR “bacterial therapeutics”)
o Bacteriophages: (“bacteriophage therapy” OR “phage modulation” OR “phage*“)
o Engineered yeast: (“engineered yeast” OR “*Saccharomyces cerevisiae*” OR “yeast*” OR “saccharomycete* “)
o Outer membrane vesicles (OMVs): (“outer membrane vesicles” OR “OMVs” OR “bacterial vesicles” OR “vesicle*”)

- Mechanistic Terms: (“synthetic gene circuits” OR “CRISPR” OR “TALEN” OR “ZFN” OR “quorum sensing” OR “immunomodulation”)

Boolean operators (AND/OR) optimized retrieval. Filters included: English language, publication years 2000–2024 (prioritizing 2018–2024 for rapid technological advances), and article types (original research, reviews, clinical trials).

Inclusion and Exclusion Criteria

- Inclusion:

o Studies on synthetic biology tools (e.g., CRISPR, TALEN) applied to microbiome engineering.
o Preclinical/clinical studies of engineered microbes (bacteria, yeast, phages, OMVs) for IBD therapy.
o Key mechanistic insights into gut microbiome-immune interactions in IBD.
o High-impact reviews (>50 citations) framing foundational concepts.

- Exclusion:

o Studies unrelated to IBD or microbiome engineering.
o Articles focused solely on diagnostics without therapeutic translation.
o Low-evidence publications (e.g., editorials without data).

### 2.2 Screening and selection process

Initial searches yielded >1,200 publications. Titles/abstracts were screened for relevance to four thematic pillars:

1. Design of engineered strains.
2. Bacteriophage modulation.
3. Yeast-based therapeutics.
4. OMVs as delivery systems.

Full texts of 328 articles were assessed. 198 references were retained based on:

- Impact: Priority to high-citation papers and recent breakthroughs.
- Thematic Coverage: Balance across engineering strategies (bacteria/phages/yeast/OMVs) and mechanistic depth.
- Critical Appraisal: Emphasis on studies with robust models (e.g., gnotobiotic mice, human microbiota transplants) and translational validation.

### 2.3 Data synthesis and limitations

As a critical narrative review, this work synthesizes evidence thematically rather than via meta-analysis. Key claims are supported by primary data from cited references.

- Limitations: PubMed-centric search may omit niche engineering studies; non-English articles were excluded. Recent preprints were incorporated where peer-reviewed.
- Bias Mitigation: Cross-referencing seminal reviews (e.g., Riglar and Silver, 2018; Cubillos-Ruiz et al., 2021) ensured coverage of landmark studies.

### 2.4 Review type clarification

This is a comprehensive narrative review with critical appraisal, not a systematic/scoping review. It emphasizes:

- Mechanistic Innovation: e.g., CRISPR-based editing, closed-loop yeast circuits.
- Therapeutic Translation: Clinical challenges and emerging solutions.

## 3 Design of engineered therapeutic strains

### 3.1 Synthetic biology

The advancement of synthetic biology enables the development of genetically engineered microbial therapies (Cubillos-Ruiz et al., 2021). While these tools permit the construction of diagnostic-therapeutic circuits where bacteria sense biomarkers (e.g., TNF-*α*), process signals via genetic logic gates (AND/OR), and deliver effectors (e.g., anti-inflammatory cytokines) their application to IBD faces significant translational barriers (Pedrolli et al., 2019; Tanna et al., 2021; Nandagopal and Elowitz, 2011; Kobayashi et al., 2004). Circuit instability under dynamic gut conditions (pH fluctuations, microbiota competition), safety concerns regarding off-target effects or horizontal gene transfer, and limited clinical validation in human studies remain critical limitations (Claesen and Fischbach, 2015). To bridge preclinical advances to therapeutic translation, future designs must incorporate IBD-specific features such as fail-safe self-destruction mechanisms and mucosa-targeting delivery systems, rather than presenting generalized technical overviews.

### 3.2 Genome edition for engineered bacteria

**Figure 2:**
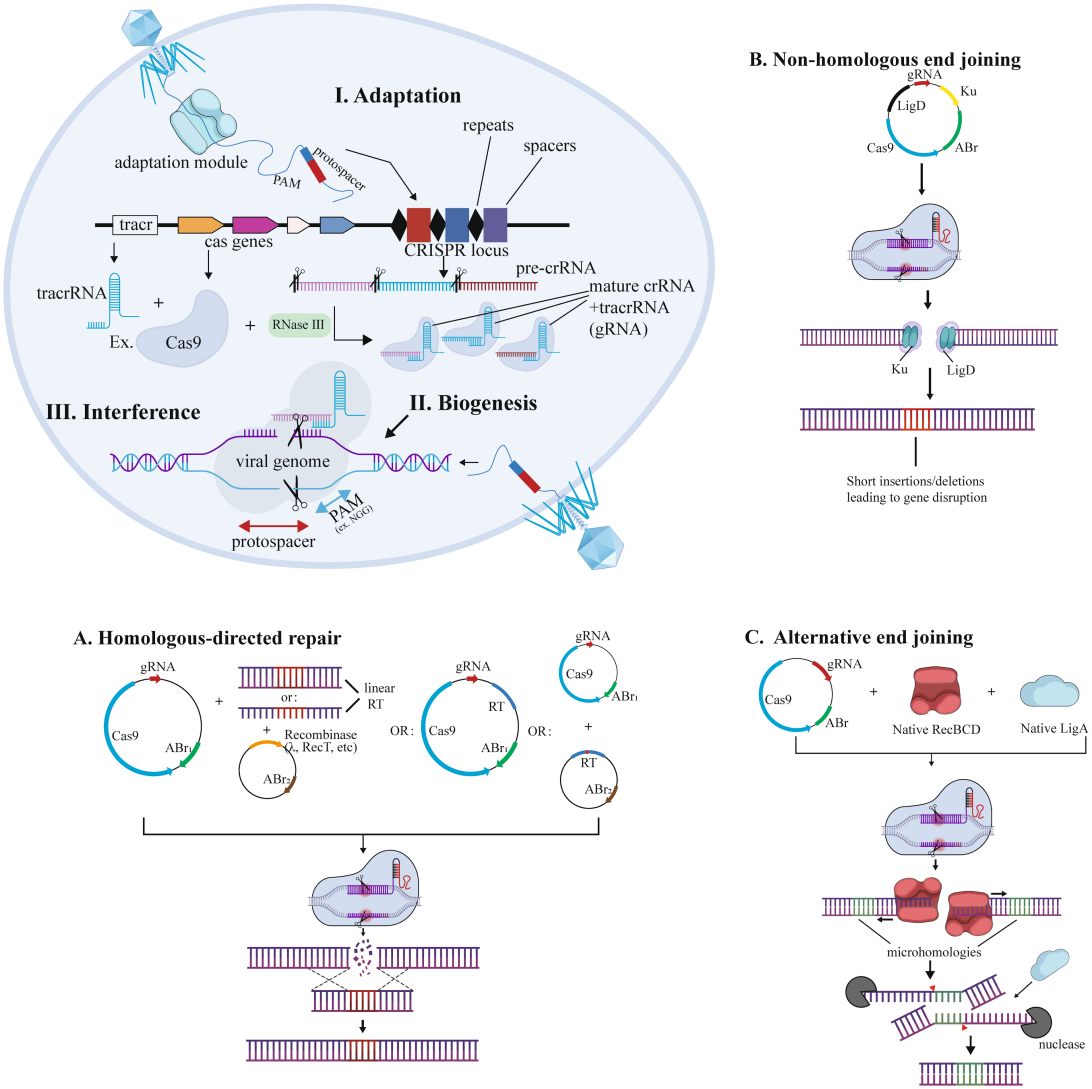
CRISPR-Cas technology for genome editing in bacteria. Editing was performed by **(a)** NHEJ (non-homologous end joining), **(b)** HDR (homology-directed repair), and **(c)** alternative end joining.

Selecting suitable microbial chassis (e.g., mucus-adherent Bacteroides or immunomodulatory *L. lactis*) and editing tools is essential for IBD therapy (Table 1; Claesen and Fischbach, 2015; Watterlot et al., 2010). As summarized in Table 2, Zinc Finger Nucleases (ZFNs) offer moderate delivery efficiency but suffer from low tolerance to non-G-rich sequences; TALENs provide high specificity yet require thymine at target sites and face delivery challenges due to size; while CRISPR-Cas systems dominate with modular design and efficiency despite PAM dependency and off-target risks (Li et al., 2020; Kim and Kim, 2014). Figure 2 illustrates how CRISPR enables genome editing. For IBD, CRISPR’s multiplex editing capacity is advantageous but requires optimization to minimize off-target effects in commensal bacteria (Kaniecki et al., 2018; Verma and Greenberg, 2016; Chang et al., 2017), underscoring the need for tool-specific adaptation rather than generic technical descriptions.

**Table 2:** Comparison of genome-editing tools for IBD microbial engineering.

Tool	Precision	Efficiency	IBD applicability	Key limitations
ZFNs	Moderate	Low	Limited by G-rich sequence requirements	Complex protein engineering needed (Riglar and Silver, 2018)
TALENs	High	Moderate	Suitable for large inserts (e.g., IL-35)	Size limits viral delivery (Riglar and Silver, 2018)
CRISPR-Cas9	High	High	Preferred for multiplexed edits (e.g., ROS-scavenging enzymes)	PAM sequence dependency (Riglar and Silver, 2018)

*C*ritique: CRISPR-Cas9 is optimal for IBD due to multiplexed editing (e.g., *L. casei* MnSOD + *Bifidobacterium* IL-10; Leventhal et al., 2020 Zaiss et al., 2021), but off-target effects risk unintended immune activation (Mager et al., 2020 Charbonneau et al., 2020). TALENs are viable for eukaryotic chassis (e.g., *S. boulardii*) but suffer from low throughput (Riglar and Silver, 2018).

### 3.3 Targeting therapeutic delivery

Precise localization to inflamed mucosa and sustained persistence constitute major translational hurdles for IBD therapies. Motility engineering (e.g., chemotaxis toward AHL signals) may fail in dysbiotic IBD guts with distorted metabolite gradients, while synthetic adhesins (e.g., INP-fused proteins) show promise but lack validation in human inflamed tissue (Van Houdt et al., 2007; Ho et al., 2018). Although *Bacteroides* spp. naturally colonize mucus layers (Esposito et al., 2021; Barrett, 2010), enhancing their persistence requires IBD-optimized strategies: Surface modifications (e.g., alginate encapsulation) risk impairing host-microbe crosstalk; genetic overexpression of adhesion factors (e.g., BINDs) could trigger immune activation (Anselmo et al., 2016; Hou et al., 2021; Gunzburg et al., 2020; Nguyen et al., 2014; Duraj-Thatte et al., 2019).; and niche competition via nutrient engineering faces instability due to IBD dietary variability (Shepherd et al., 2018; Celebioglu et al., 2017). Critically, the absence of clinical data on engineered strains in colitis models and scalability challenges for personalized formulations highlight the gap between preclinical advances and therapeutic reality.

## 4 Microbiome modulation by bacteriophages

The human microbiome, composed of trillions of microorganisms inhabiting diverse anatomical sites, plays a critical role in maintaining health and homeostasis. Bacteriophages, or phages, represent an essential component of this intricate ecosystem, significantly influencing the composition, diversity, and functional dynamics of microbial communities. A comprehensive understanding of the mechanisms by which bacteriophages modulate the microbiome is pivotal for harnessing their therapeutic potential in addressing various diseases, including IBD (Dion et al., 2020). Dysbiosis of the gut microbiota has been closely linked to the pathogenesis and progression of IBD (Sinha et al., 2022). The gut virome, predominantly consisting of bacteriophages, is recognized as a critical regulator of gut microbiota composition and function (Sinha et al., 2022).

Bacteriophages are viruses characterized by their specific tropism for infecting and replicating within bacterial hosts (Dion et al., 2020). Through these interactions, phages can profoundly reshape microbial communities and impact ecosystem stability and host health by altering microbial diversity and abundance (Dion et al., 2020).

The role of bacteriophages in regulating gut homeostasis and disease pathogenesis is an active area of research, with observed alterations in phage composition during disease progression (Federici et al., 2023). Under healthy conditions, the gut virome is characterized by a stable, long-term community structure, dominated by crAss-like and *Microviridae phages*, which constitute the majority of intestinal viruses (Shkoporov et al., 2019). These phages are closely associated with specific bacterial taxa and contribute to maintaining gut microbiota equilibrium (Shkoporov et al., 2019; Cornuault et al., 2018). In contrast, in IBD, this equilibrium is disrupted, resulting in significant alterations to the gut virome (Clooney et al., 2019). For example, in patients with active ulcerative colitis (UC), an overrepresentation of temperate phages has been linked to a reduction in *Bacteroides thetaiotaomicron* and *Bacteroides uniformis* (Nishiyama et al., 2020). Furthermore, studies report altered abundance of *Caudovirales* in IBD, which is associated with reduced bacterial diversity and exacerbated colitis in models (Wagner et al., 2013; Zuo et al., 2019). Gut inflammation is hypothesized to trigger the induction of prophages into the lytic cycle, thereby destabilizing the phage community (Clooney et al., 2019). Additionally, an increased abundance of *Caudovirales* phages has been observed in IBD patients, correlating positively with disease severity (Zuo et al., 2019). These findings highlight the dynamic nature of the gut virome during health and disease, underscoring its critical relationship with gut microbiota structure and disease pathogenesis.

All pathogenic bacteria associated with the progression of IBD represent potential targets for phage combination therapy. For instance, studies in a susceptible mouse model of ulcerative colitis (UC), an IBD-related model, demonstrated that a phage combination effectively suppressed *Klebsiella pneumoniae* and attenuated its induction of proinflammatory responses (Federici et al., 2023; Kitamoto et al., 2020). Moreover, genetic engineering can expand the host range of phages. For example, phages originally targeting *Escherichia coli* have been engineered to infect *Yersinia* and *Klebsiella* species, and vice versa, through the modification of their tail fibers (Ando et al., 2015). However, a limitation of phage therapy is the potential emergence of resistance mutations, comparable to antibiotic therapy (Dedrick et al., 2019). To address this issue, the use of phage combinations, where each phage employs distinct mechanisms to infect target bacteria, can delay resistance development and exert longer-term suppressive effects (Wright et al., 2019). Additionally, phage therapy exhibits immunomodulatory potential. Elevated phage levels have been shown to induce interferon-*γ* (IFN-γ) secretion, mediated by toll-like receptor 9 (TLR9) in mouse models and human cells (Shuwen and Kefeng, 2022). This immunomodulatory effect suggests phage therapy might function as a tolerogenic strategy for UC, as proposed based on preclinical findings (Shuwen and Kefeng, 2022; Gogokhia et al., 2019).

Phage therapy offers several significant advantages. First, it can delay the development of bacterial resistance (Nikolich and Filippov, 2020). Through the design of diverse phage combinations, it is possible to suppress multiple strains and species of pathogens while reducing the likelihood of treatment resistance emergence, as each phage targets bacteria through distinct mechanisms (Dedrick et al., 2019). The second advantage is specificity and self-replication of phages. Phages have narrow host specificity, allowing them to selectively target pathogenic bacteria without disrupting the surrounding microbial community (Federici et al., 2022). Furthermore, the ability of phages to self-replicate within host bacteria ensures sustained therapeutic efficacy when target pathogen levels exceed a critical threshold (Federici et al., 2022). The selection of strictly lytic bacteriophages, or the genetic modification of natural bacteriophages through the deletion of integrase genes or the alteration of their specificity to pre-identified hosts, can enhance bacterial lysis efficiency while minimizing the risk of horizontal gene transfer of toxins or antibiotic resistance genes into bacterial chromosomes via lysogeny (Pires et al., 2021). The third advantage is the feasibility of oral administration. Orally administered phages can accumulate in the gastrointestinal tract, particularly in the lower gut and fecal matter. This administration route avoids immunogenic reactions associated with systemic delivery, thereby improving treatment acceptability (Majewska et al., 2019). Furthermore, encapsulation of phages in materials such as alginate, polyethylenimine, and pectin enables controlled release in the lower gastrointestinal tract, optimizing oral delivery efficacy while reducing potential physiological disruptions (Hsu et al., 2020). In summary, phage therapy represents a promising therapeutic strategy, providing precise targeting of specific pathogens, mitigating the risk of bacterial resistance development, and offering broad potential applications in microbiome research.

While preclinical evidence, particularly from IBD-relevant models like UC, supports the potential of phage therapy for IBD, critical evaluation reveals gaps (Federici et al., 2023; Kitamoto et al., 2020). Many mechanistic insights linking phage dysbiosis to IBD stem from association studies, necessitating further causal investigation in relevant models (Clooney et al., 2019; Zuo et al., 2019). The promising immunomodulatory effects observed require validation in the complex inflammatory milieu of human IBD (Shuwen and Kefeng, 2022). Furthermore, robust clinical data demonstrating efficacy and safety of phage cocktails specifically in IBD patients are currently lacking (Federici et al., 2022; Federici et al., 2023). Challenges such as the rapid evolution of phage resistance, potential immunogenicity upon repeated dosing, and the need for standardized, personalized phage cocktail formulations remain significant hurdles for clinical translation in IBD (Dedrick et al., 2019).

## 5 Engineered yeast

Engineered yeast represents a distinct and promising therapeutic modality for IBD, leveraging its eukaryotic cellular machinery and genetic tractability for sophisticated engineering (Mimee and Nagler, 2021). Currently, engineered yeast is employed to modulate dysregulated purinergic signaling, a key feature of IBD pathogenesis. Scott *et al*. investigated the enzymatic conversion of extracellular ATP (eATP) into immunosuppressive adenosine as a potential strategy to disrupt the inflammatory cycle (Scott et al., 2021). However, excessive adenosine signaling can lead to adverse effects, such as fibrosis and tissue destruction, and a delicate imbalance between eATP and adenosine levels in the gut (Mimee and Nagler, 2021; Scott et al., 2021). To address this issue, Scott et al. developed a closed-loop therapeutic system using engineered yeast. They designed a transcriptional biosensor in *Saccharomyces cerevisiae* to detect eATP levels associated with inflammation and connected it to a secreted potato apyrase enzyme capable of degrading eATP (Mimee and Nagler, 2021; Scott et al., 2021). In mouse models of chemically induced colitis, this closed-loop system demonstrated superior efficacy compared to open-loop designs, specifically evidenced by reduced inflammation, mitigated tissue fibrosis, and ameliorated dysbiosis, underscoring its therapeutic potential (Mimee and Nagler, 2021; Scott et al., 2021).

Significant progress has also been made in engineering yeast strains for sustained butyrate production to combat intestinal inflammation. Butyrate, a crucial short-chain fatty acid produced by fibrolytic bacteria, exhibits immunomodulatory properties and promotes the proliferation of regulatory T cells (Tregs) in the intestinal mucosa (Kong et al., 2024). Recent studies have engineered brewer’s yeast (*Saccharomyces cerevisiae*) to serve as an efficient butyrate producer (Wu et al., 2024). The engineering process involves several key steps. First, genes essential for butyrate production in various hosts are identified and codon-optimized for yeast, followed by the synthesis of these gene sequences. Subsequently, these genes are introduced into yeast cells *via* plasmid vectors, which are integrated into the *S. cerevisiae* genome to enable gene expression (Wu et al., 2024). To enhance butyrate production under anaerobic conditions, researchers introduced metabolic modules, including acetoacetyl-CoA enhancement, acetyl-CoA enhancement, NADH enhancement, and acyl-CoA regulation modules. These modifications enabled the engineered yeast to sustain butyrate production in the intestinal environment, ensuring consistent therapeutic efficacy (Wu et al., 2024). Experimental results demonstrated that strains with moderate butyrate production levels exhibited the most pronounced therapeutic effects. Furthermore, synthetic biology approaches provided mechanisms for butyrate release in response to disease-specific signals, potentially improving therapeutic outcomes. Engineered yeast can autonomously regulate butyrate production based on environmental butyrate concentrations, enabling precise and controlled therapeutic dose delivery (Wu et al., 2024).

However, critical translational challenges persist beyond proof-of-concept efficacy. Engineered yeast strains exhibit transient gut colonization (detectable for ≤48 h post-administration) and lack sustained engraftment, necessitating frequent dosing that may compromise patient compliance in chronic IBD management (Scott et al., 2021; Wu et al., 2024). Immunogenicity risks remain underexplored, as repeated exposure to engineered eukaryotic chassis (e.g., expressing heterologous enzymes like apyrase or bacterial butyrate-pathway genes) could provoke host immune responses, including neutralizing antibodies or unintended inflammation (Scott et al., 2021; Wu et al., 2024). Long-term safety assessments are limited by short-duration preclinical studies (typically ≤7 days), leaving gaps in understanding chronic toxicity, genomic instability, horizontal gene transfer, or ecological disruption of commensal mycobiota (Scott et al., 2021; Wu et al., 2024).

Additionally, the role of yeast in modulating mucosal immunity, particularly via IgA, provides another therapeutic avenue. It has been found that dysbiosis in the gut microbiota leads to impaired immune function, characterized by atrophy of lymphoid organs and decreased levels of immunoglobulin A (IgA; Díaz-Garrido et al., 2021). Secretory immunoglobulin A (sIgA) antibodies are widely regarded as critical regulators of intestinal homeostasis, serving as the primary defense mechanism against invasive pathogens, toxins, and harmful dietary or bacterial metabolites (Conrey et al., 2023). Previous studies have shown that sIgA exhibits broad cross-reactivity with various bacterial species (Doron et al., 2021). Furthermore, *Candida albicans* and its hyphal form have been identified as key targets and potent inducers of antifungal sIgA responses. These findings suggest potential for engineering non-pathogenic yeast strains (e.g., *S. cerevisiae*) to modulate sIgA responses beneficially in IBD, although this concept requires direct experimental validation in disease models (Doron et al., 2021).

In conclusion, engineered yeast offers unique advantages for IBD therapy, including sophisticated eukaryotic gene regulation circuits and potentially lower endotoxin concerns compared to some bacterial platforms (Cubillos-Ruiz et al., 2021). Substantial progress has been made in developing systems for eATP/adenosine modulation and butyrate production. Nevertheless, significant challenges persist beyond the core engineering achievements. These include ensuring reliable long-term colonization and engraftment of engineered strains within the competitive gut niche, comprehensively assessing potential immunogenicity upon repeated administration, establishing long-term safety profiles in humans, and fine-tuning therapeutic windows to maximize efficacy while minimizing off-target effects (Mimee and Nagler, 2021; Scott et al., 2021; Wu et al., 2024). Furthermore, direct comparisons of delivery efficiency, control precision, and therapeutic efficacy between engineered yeast, bacteria, and phage-based approaches within relevant IBD models are needed to fully define their respective niches (Federici et al., 2022; Federici et al., 2023; Mimee and Nagler, 2021). Despite these hurdles, synthetic biology tools continue to provide exciting avenues for developing personalized yeast-based treatments and optimizing therapeutic outcomes for IBD.

## 6 Engineered bacteria out-membrane nanovesicles

**Figure 3:**
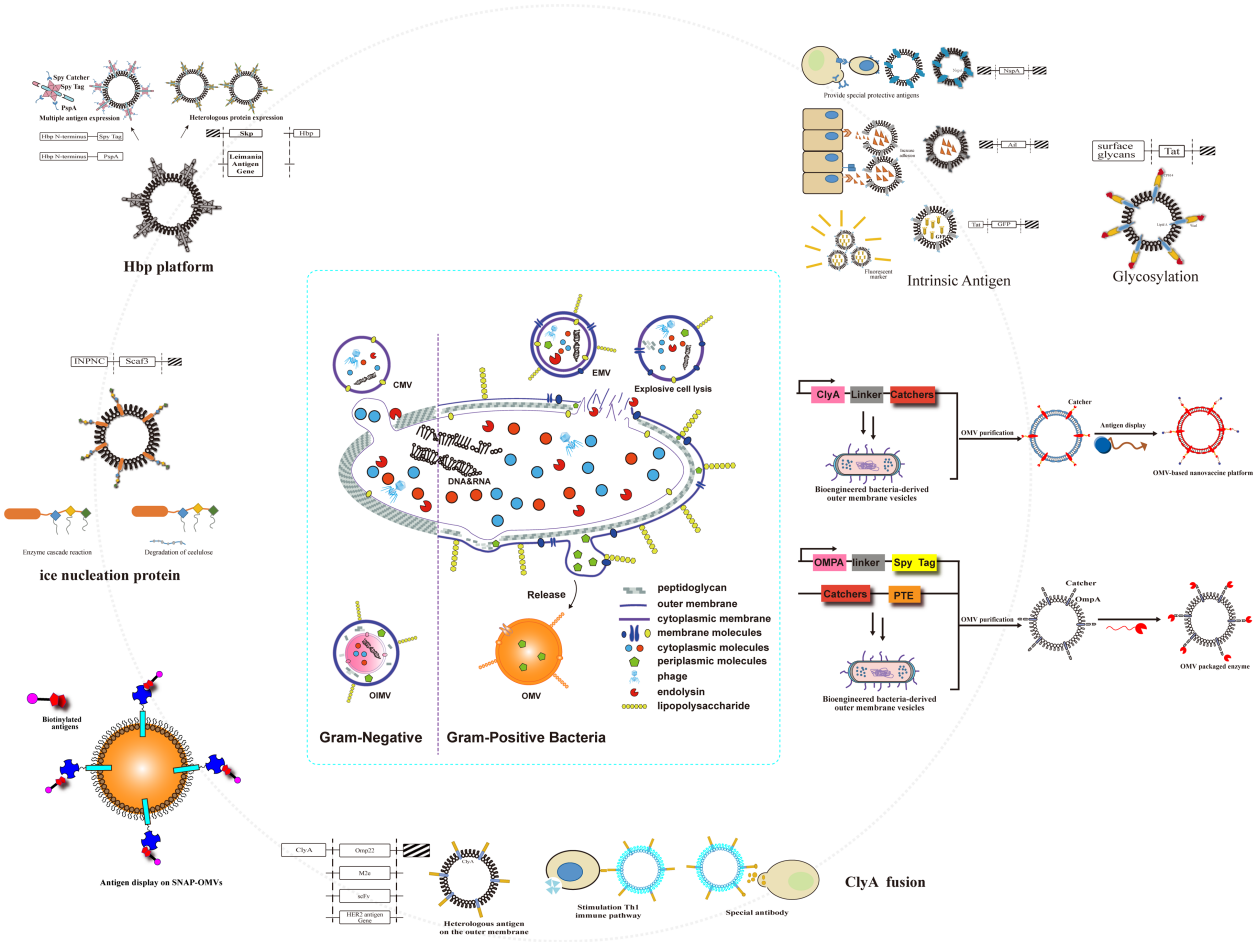
Genetic engineering modified naturally occurring OMVs to obtain a versatile transformation platform. Engineering approaches included fusion of several transmembrane proteins on OMVs; spy tags/spy traps; Hbp platforms; intrinsic antigens; glycosylation; and ice nucleoprotein fusions.

IBD therapeutics have witnessed significant advancements in recent years, with bacterial outer membrane vesicles (OMVs) emerging as a promising therapeutic strategy (Toyofuku et al., 2023; Toyofuku et al., 2019; Guerrero-Mandujano et al., 2017; Sartorio et al., 2021; Figure 3). OMVs, nano-scale (20–250 nm) extracellular vesicles constitutively released by both Gram-negative and Gram-positive bacteria, mediate important interactions within the intestinal microenvironment through intercellular and cross-species communication, thereby maintaining intestinal homeostasis (Toyofuku et al., 2019; Pegtel and Gould, 2019; Jiang et al., 2016; Chu et al., 2016). These vesicular structures exhibit distinct biological properties, including membrane protein enrichment (e.g., phospholipids, glycoproteins) and cargo molecule encapsulation (e.g., virulence factors, nucleic acids), which differ substantially from their parental bacterial cells (Toyofuku et al., 2023; Toyofuku et al., 2019). OMV biogenesis occurs through two distinct mechanisms: membrane blebbing, characterized by the formation and fission of outer membrane protrusions (Toyofuku et al., 2019; Turnbull et al., 2016), and endolysin-mediated cell lysis, which is triggered under environmental stress conditions (e.g., DNA damage) via enzymatic degradation of peptidoglycan layers (Toyofuku et al., 2019; Brown et al., 2015). Their inherent biomimetic properties, including cell membrane permeability and structural stability, enable OMVs to translocate across biological barriers and deliver functional cargo to recipient cells (Toyofuku et al., 2023; Toyofuku et al., 2019; Kulp and Kuehn, 2010). This delivery capability highlights the potential of OMVs as versatile nanoplatforms for targeted drug delivery and immunomodulation in IBD management (Toyofuku et al., 2019; Turnbull et al., 2016).

Within the host-microbe interactome, OMVs mediate important biological functions through two principal mechanisms. First, OMVs mediate horizontal gene transfer (HGT) by delivering bacterial genetic cargo (e.g., genomic DNA, non-coding RNAs) to eukaryotic cells, a process conserved across diverse bacterial taxa (Tashiro, 2017; Mills et al., 2024; Tran and Boedicker, 2017). This vesicle-facilitated nucleic acid transport induces host epigenetic reprogramming *via* RNA-mediated transcriptional modulation, though the molecular basis of vesicle internalization remains mechanistically unresolved, which may involve receptor-mediated competitive uptake (Toyofuku et al., 2023). Such HGT proficiency underscores their potential as tools for microbial genome engineering.

Second, OMVs harbor bacteriolytic enzymes (e.g., glycoside hydrolases) and antimicrobial metabolites capable of lysing competing microbiota (Kadurugamuwa and Beveridge, 1996; Yue et al., 2021). This innate antimicrobial activity, combined with engineered cargo encapsulation, positions OMVs as targeted therapeutics for IBD management. Specifically, synthetic OMV formulations could selectively deplete pro-inflammatory pathobionts while preserving commensal symbionts, thereby rectifying intestinal dysbiosis - a central pathogenic driver in IBD.

Beyond their role in microbial communication, OMVs function as potent immunomodulators through pathogen-associated molecular pattern (PAMP) recognition (Kaparakis-Liaskos and Ferrero, 2015). Specifically, OMVs-associated ligands activate an array of pattern recognition receptors (PRRs) on innate immune cells, eliciting cytokine release, inflammasome activation, and apoptotic cascade initiation (Kaparakis-Liaskos and Ferrero, 2015; Söderblom, 2005). Notably, OMVs exhibit bidirectional immunoregulatory activity - exacerbating or attenuating inflammatory responses *via* PRR engagement, while concurrently transferring non-coding RNAs that post-transcriptionally regulate host immune gene networks (Cañas et al., 2018; Gilmore et al., 2022). Of translational significance, probiotic-derived OMVs mediate calibrated immune stimulation, preserving intestinal immune equilibrium through TLR ligand exposure (Shen et al., 2012). *Bacteroides fragilis* OMVs encapsulate polysaccharide A (PSA), which activates TLR2/4-dependent signaling to dampen hyperactive immunity while enhancing commensal microbiota colonization (Mazmanian et al., 2008; Molina-Tijeras et al., 2019; Rothfield and Pearlman-Kothencz, 1969). This supports a novel therapeutic hypothesis for IBD.

Emerging evidence highlights the immunomodulatory role of probiotic-derived OMVs in maintaining intestinal homeostasis (Shen et al., 2022). Specifically, *Bacteroides fragilis* OMVs activate TLR2 signaling in dendritic cells (DCs), resulting in the induction of regulatory T cell (Treg) differentiation and the production of interleukin-10 (IL-10), thereby ameliorating 2,4,6-trinitrobenzene sulfonic acid (TNBS)-induced colitis in rodent models (Chu et al., 2016; Shen et al., 2012). Mechanistically, OMVs-DC interactions upregulate IL-10 expression through the IBD-associated autophagy gene ATG16L1, suppressing intestinal inflammation in preclinical models (Chu et al., 2016; Durant et al., 2020). Furthermore, administration of *Bacteroides fragilis* OMVs was shown to ameliorate dextran sulfate sodium (DSS)-induced colitis in mice, reducing disease activity and histological damage, further supporting their therapeutic potential in IBD-relevant models (Durant et al., 2020). Notably, the abundance of probiotic species (e.g., *Bacteroides fragilis*) is markedly reduced in IBD patients, suggesting that OMVs-mediated immunoregulation primarily operates in healthy physiological states (Durant et al., 2020). Intriguingly, *Bacteroides thetaiotaomicron* (Bt)-derived OMVs (BEVs) exhibited state-dependent immunomodulation: in healthy conditions, BEVs enriched cycling monocytes and maintained tissue-resident macrophage pools (Swirski et al., 2014). However, BEV proteins enhanced DNA repair in monocytes, potentially mitigated oxidative DNA damage linked to colorectal carcinogenesis in UC (Liao et al., 2008). Furthermore, BEVs modulated the unfolded protein response (UPR) in inflammatory monocytes by promoting apoptosis and endoplasmic reticulum-associated degradation (ERAD), thereby alleviating ER stress and attenuating intestinal inflammation (Jones et al., 2018). These findings underscored the therapeutic potential of exogenous OMVs supplementation in IBD management (Shen et al., 2022).

In the field of synthetic biology, OMVs are mainly applied as vaccine delivery platforms and drug delivery systems (Sartorio et al., 2021; Gnopo et al., 2017; Carvalho et al., 2019; Elhenawy et al., 2014). By fusing exogenous antigens with OMVs-enriched proteins, such as ClyA, these antigens are more readily transported into the periplasmic space and subsequently packaged into the OMVs lumen (Gnopo et al., 2017; Wai et al., 2003; Chen et al., 2010). This capability enables OMVs to carry multiple antigens and elicit specific antibody responses, thereby conferring protection against pathogenic microorganisms (Sartorio et al., 2021). Engineered bacterial strains can produce OMVs loaded with therapeutic proteins or drugs, which serve as efficient delivery vehicles for transporting these agents to targeted sites (Sartorio et al., 2021; Carvalho et al., 2019). A critical limitation in such applications stems from the inherent self-toxicity of OMVs. To mitigate this challenge, two principal strategies have been developed through rigorous investigation (Gnopo et al., 2017). The first one is to modify the structure of lipopolysaccharides (LPS). Techniques include reducing acyl chain numbers or converting to monophosphorylated lipid A, resulting in detoxified OMVs (Gnopo et al., 2017; Chen et al., 2016; Needham et al., 2013; Irene et al., 2019). The other is to edit the bacterial gene related to LPS expression. Genetic engineering can control LPS synthesis pathways, producing OMVs with reduced immune system activation and adverse effects (Gnopo et al., 2017).

There are many mysteries about OMVs yet to be revealed, including its formation process and mechanisms about nucleic acid packaging. In addition, OMVs are usually purified from bacteria cultured under standard laboratory conditions, but their composition may differ in wild type strains. Nevertheless, with the development of research on OMVs, the therapeutic potential of OMVs for IBD is gaining more and more attention (Sartorio et al., 2021).

## 7 Perspective and future developments

### 7.1 Technical challenges

**Figure 4:**
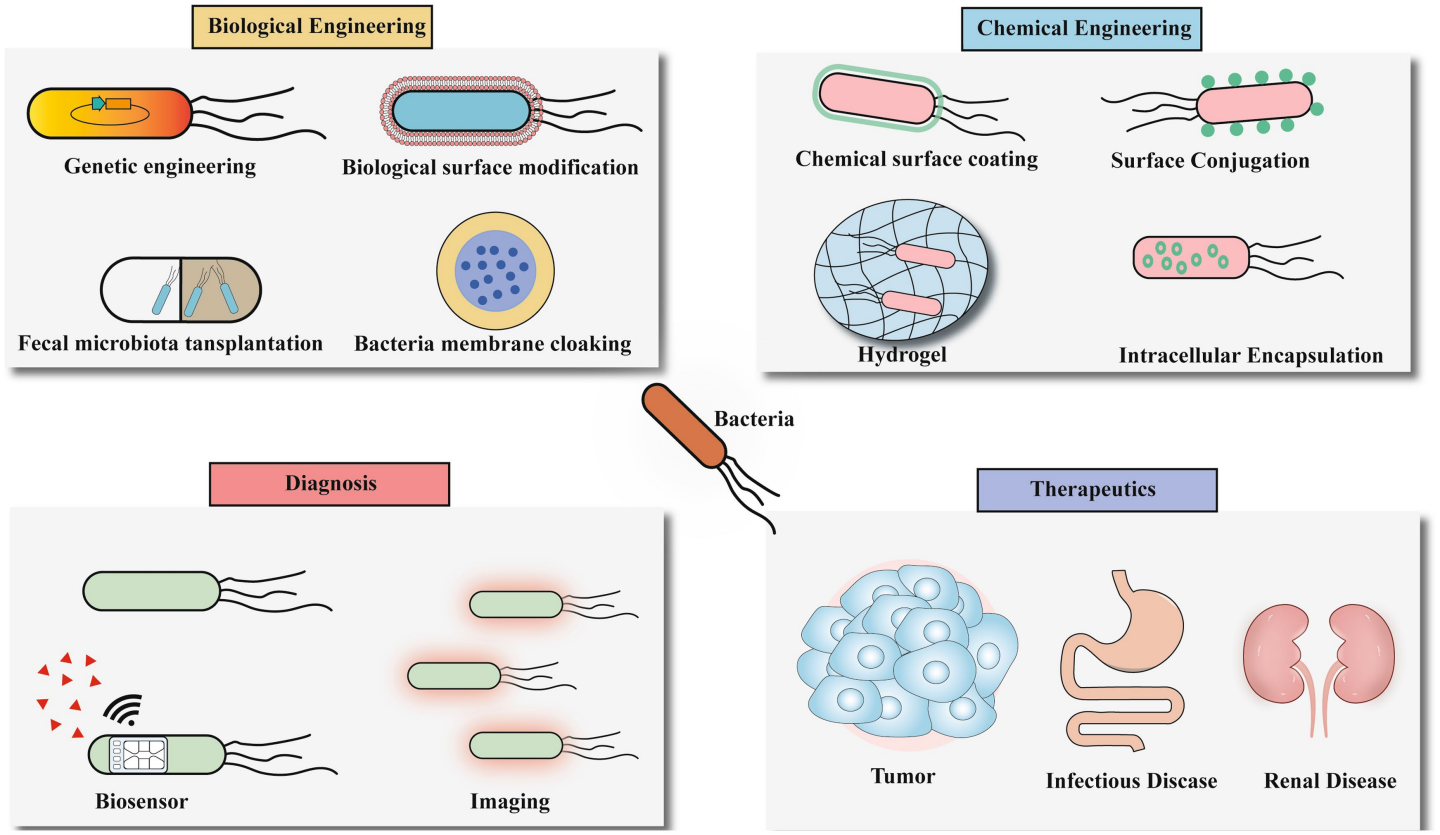
The future application of microbiome engineering.

Bacteria serve as pivotal platforms in microbiome engineering, demonstrating remarkable potential for IBD therapeutics through genetic modification, surface engineering, and targeted delivery approaches (Figure 4). These engineered bacterial systems offer unprecedented precision in gut microbiota modulation, paving the way for next-generation personalized microbial therapies. However, translating this potential into clinical reality faces significant technical hurdles.

Engineering Complexity: microbiome engineering seeks to modulate the composition or activity of microbial communities, the extraordinary diversity and structural complexity of these ecosystems present formidable barriers to targeted manipulation (Marsh and Ley, 2022). This challenge is compounded by the prevalence of unculturable species under standard laboratory conditions and species-specific variations in DNA uptake mechanisms and integration pathways that hinder horizontal gene transfer (Marsh and Ley, 2022; Jin et al., 2022; Waller et al., 2017). Furthermore, microbial defense systems (e.g., restriction-modification, phage exclusion) act as evolutionary safeguards against foreign genetic material, necessitating species-tailored engineering approaches (Bernheim and Sorek, 2020). Current methodologies remain predominantly optimized for individual microbial taxa rather than complex consortia (Marsh and Ley, 2022; Johnston et al., 2019).

Circuit Burden and Genetic Stability: Engineered microbes sense, memorize, and respond to biological signals through synthetic circuits (Riglar and Silver, 2018). However, the “circuit burden”—the metabolic load or cost incurred—can impact cell stability, mutation rates, and lead to loss of function (Ceroni et al., 2015). This necessitates establishing an optimal equilibrium between circuit functionality and metabolic load, presenting substantial design and implementation challenges (Pedrolli et al., 2019). Successful implementation requires synergistic integration of biosensors, intracellular logic processors, and effectors, but biological components exhibit limited orthogonality, mandating dedicated circuit architectures per cellular unit to prevent cross-talk (Pedrolli et al., 2019). Regarding genetic stability optimization, contemporary therapeutic strains predominantly utilize chromosomal integration of recombinant DNA to ensure heritable stability. Notably, plasmid-based systems demonstrate inherent instability due to segregational loss under non-selective conditions and unequal partitioning during cytokinesis (Hwang et al., 2017). Current stabilization strategies include: essential gene complementation systems involving chromosomal deletion paired with plasmid-borne rescue cassettes, and advanced maintenance mechanisms employing toxin-antitoxin modules and plasmid partition proteins for longitudinal plasmid persistence (Ho et al., 2018).

**Figure 5:**
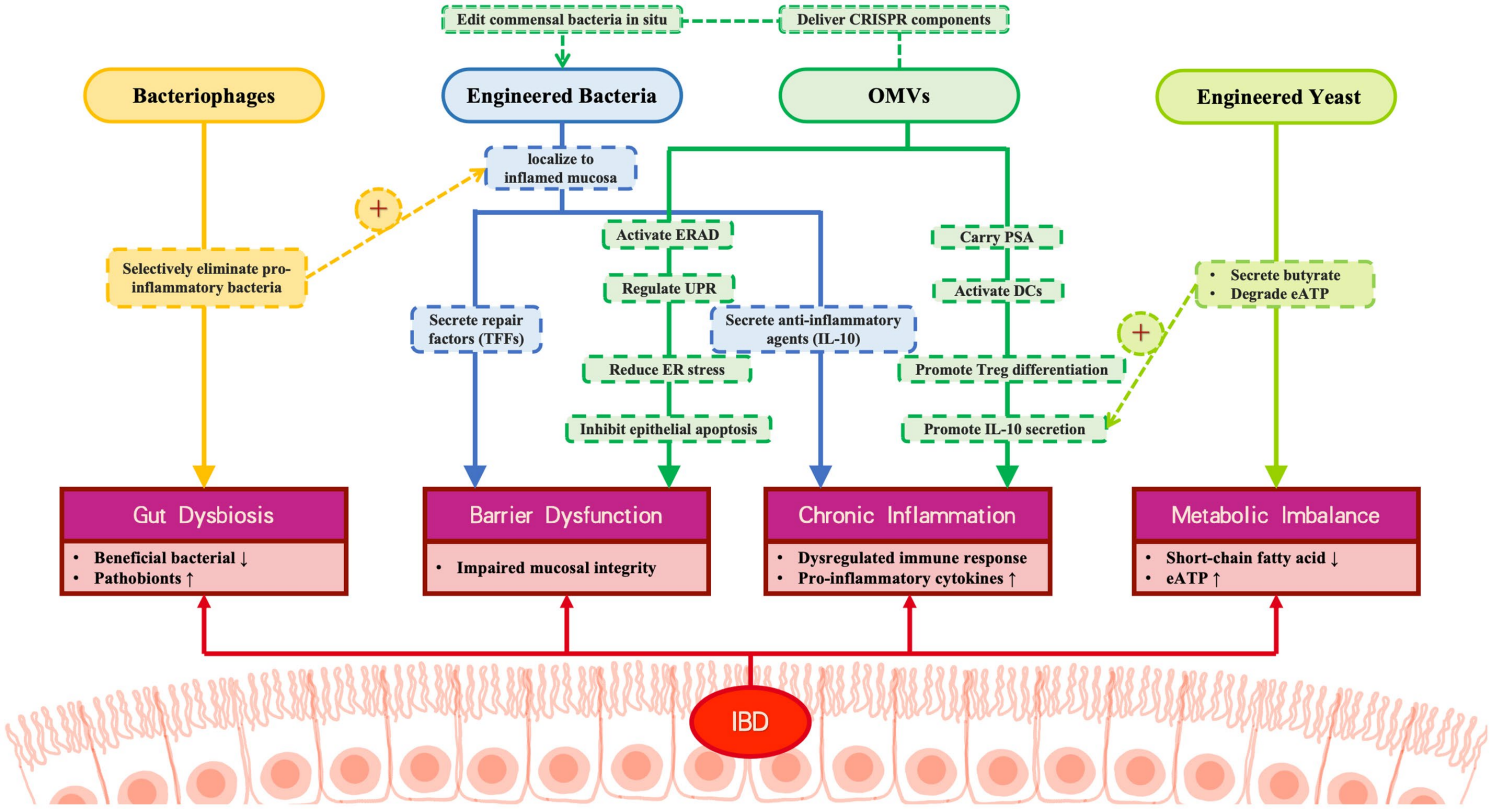
Multi-modal engineered biotherapeutic system for IBD intervention. This figure illustrates the synergistic therapeutic mechanism of four engineered biological components for IBD: Bacteriophages: Selectively eliminate pro-inflammatory bacteria, correcting microbial dysbiosis; Engineered bacteria: 1. Secrete repair factors (TFS) to inhibit epithelial apoptosis. 2. Secrete anti-inflammatory agents (IL-10). Engineered yeast: Produce butyrate and degrade the pro-inflammatory factor eATP, alleviating metabolic imbalance; OMVs: 1. Deliver PSA to activate immune tolerance. 2. Restore barrier function via the ERAD/UPR pathway. 3. Deliver CRISPR components to engineered bacteria.

Delivery System Limitations: While viral vectors (retrovirus, lentivirus, adenovirus, AAV) offer high delivery efficiency, safety concerns like immunogenicity and insertional mutagenesis persist (Maggio et al., 2014). Nonviral systems (electroporation, hydrodynamic injection, lipid nanoparticles) provide biocompatibility but often suffer from reduced efficacy in vivo (Li et al., 2020). To address these limitations, optimization strategies include hybrid viral vectors (e.g., PEGylated adenovirus/AAV (Zou, 2022), gold nanoparticle-polymer hybrids; Lee et al., 2017), structural modifications (e.g., minicircle DNA; Kay et al., 2010), chemical conjugation, and nonviral polymer complexes (Guenther et al., 2014), collectively enhancing precision, efficiency, and safety (Koo et al., 2017; Figure 5).

Biocontainment and Safety: Beyond technical challenges, ethical and biosafety considerations constitute critical barriers. Central to this is implementing stringent biocontainment protocols during deployment to prevent horizontal gene transfer, regulate proliferation, and ensure safety (Riglar and Silver, 2018). Advanced strategies include environmentally triggered kill switches (e.g., temperature-sensitive toxin-antitoxin; Piraner et al., 2017; Mandell et al., 2015) and synthetic auxotrophy mechanisms (metabolic dependency on non-canonical amino acids/xenonucleotides (Stirling et al., 2017; Gallagher et al., 2015)). While effective at reducing escape frequencies, long-term efficacy is challenged by evolutionary pressures favoring mutation-driven resistance (Riglar and Silver, 2018). This urgently requires development of next-generation containment modules tailored for living therapeutics. Strain selection and attenuation are equally pivotal safety determinants (Brockstedt, 2004; Wallecha, 2009). Episodes of residual pathogenicity (e.g., listeriosis in CRS-207 trials) highlight the need for rigorous safety protocols (Riglar and Silver, 2018).

### 7.2 Clinical translation barriers and future research directions

Despite the promise, the effectiveness of engineered microbiome therapies in humans remains to be fully verified. Techniques for assessing *in vivo* effects lag behind gene editing advancements (Riglar and Silver, 2018). Specific challenges and future research foci vary by therapeutic modality:

Engineered Bacteria and Yeast: Key challenges include achieving stable colonization and persistent therapeutic activity in the dynamic gut environment during active inflammation (Mimee and Nagler, 2021; Wu et al., 2024), assessing potential immunogenicity upon repeated dosing, establishing long-term human safety profiles, and fine-tuning therapeutic windows (Mimee and Nagler, 2021; Scott et al., 2021; Wu et al., 2024). Future research prioritizes refining dose–response calibration, optimizing site-specific delivery (e.g., synthetic adhesins; Piñero-Lambea et al., 2015), exploring polymodal therapeutic synergies, and integrating synthetic gene networks for patient-specific regimens stratified by disease endotypes (Wu et al., 2024). Precisely modulating critical balances (e.g., extracellular ATP and adenosine; Mimee and Nagler, 2021) within narrow therapeutic windows is crucial for minimizing off-target effects. Prospectively, engineered yeast systems hold particular promise, with designs enabling sophisticated multi-input/output regulatory circuits (Mimee and Nagler, 2021).

Phage Therapy: Phage therapy encounters substantial challenges in complex IBD management (Federici et al., 2023). Primary limitations encompass rapid evolution of phage-resistant strains and unintended immunomodulatory effects. While rationally designed polyphage cocktails targeting specific pathogens (Federici et al., 2022; Dedrick et al., 2019) and computational optimization (genomic mining, machine learning; Federici et al., 2023; Thiebes et al., 2020; Yehl et al., 2019) are promising, critical unresolved issues include phage stability in the GI tract, biodistribution, and immune interactions. Elucidating phage-mediated immune modulation mechanisms is a critical priority (Shuwen and Kefeng, 2022). Combination therapies with antibiotics or FMT show synergistic potential preclinically (Eskenazi et al., 2022; Suez et al., 2018). Rigorous clinical validation through large-scale RCTs is imperative to establish pharmacodynamics and biosafety (Federici et al., 2022; Federici et al., 2023).

OMVs: Emerging evidence demonstrates the dual immunomodulatory capacity of OMVs, which can either exacerbate pathology or induce tolerance (Toyofuku et al., 2023; Kaparakis-Liaskos and Ferrero, 2015). While applications expand beyond vaccines into drug delivery and synthetic biology (Sartorio et al., 2021; Mashburn and Whiteley, 2005; Hoefler et al., 2017), significant translation hurdles persist: inherent heterogeneity complicating characterization, scalability limitations, and potential immunogenicity from endogenous components like LPS (Sartorio et al., 2021; Needham et al., 2013). These limitations drive efforts to engineer OMVs with tailored compositions. Robust clinical validation across IBD models remains imperative (Toyofuku et al., 2023).

### 7.3 Roadmap to clinical translation

Translating engineered microbiome therapies from bench to bedside for IBD patients requires a defined clinical development pathway. This sequential roadmap, outlined in Table 3, encompasses key stages from preclinical optimization through regulatory approval and post-marketing surveillance, addressing the unique challenges of these living therapeutics.

**Table 3:** Proposed clinical translation roadmap for engineered microbiome therapies in IBD.

Stage	Primary goals	Key considerations
Preclinical optimization	Safety (biocontainment, toxicity) Efficacy (models, engraftment) PK/PD, Scalable manufacturing	Sophisticated models (gnotobiotic, humanized), GMP protocols
Phase I (Safety)	Tolerability Initial safety profile Preliminary PK/PD	Small cohorts (healthy volunteers/small IBD group) Dose escalation
Phase II (Proof-of-Concept)	Efficacy signals (clinical, endoscopic, biomarkers) Optimal dosing Further safety	IBD patient populations Mechanistic substudies Biomarker validation
Phase III (Confirmation)	Confirm efficacy vs. standard care/placebo Safety in large diverse populations	Large RCTs Realistic endpoints (clinical remission, mucosal healing)
Regulatory approval & Phase IV	Submission to agencies Post-marketing surveillance	Defining clear regulatory pathways Long-term safety monitoring Real-world effectiveness

In conclusion, while significant technical and clinical challenges persist, engineered live biotherapeutics—including symbiotic bacteria, yeast consortia, phage systems, and programmable OMVs—exhibit considerable therapeutic potential for IBD. Though distinct, their strategic integration through systems biology frameworks could catalyze paradigm-shifting advances in precision microbiome therapeutics. Navigating the outlined roadmap, with focused research addressing the key barriers and a commitment to robust clinical evaluation, is essential for realizing this potential for IBD patients.

## Glossary

IBDs: Inflammatory bowel diseases
OMVs: out-membrane nanovesicles
ZFNs: Zinc Finger Nucleases
TALENs: transcription activator-like effector nucleases
CRISPR: clustered regularly interspaced short palindromic repeat-Cas-associated nucleases
DSBs: double-stranded breaks
HDR: homology-directed repair
NHEJ: non-homologous end joining
AAV: adeno-associated virus
AHL: N-acyl-L-homoserine lactone
QS: Quorum sensing
INP: ice nucleation protein
BINDs: Biofilm Integrated Nanofiber Displays
NO: nitric oxide
TFFs: trefoil factors
EcN: *Escherichia coli* Nissle
IL-35: interleukin-35
IL-10: interleukin-10
CAT: catalase
SOD: superoxide dismutase
MnSOD: manganese superoxide dismutase
SICE: Stress-Inducible Controlled Expression
ANP: atrial natriuretic peptide
PEA: paracasei F19 express
UC: ulcerative colitis
CD: Crohn’s disease
EGF: epidermal growth factor
IFN-γ: interferon-*γ*
eATP: extracellular ATP
IgA: immunoglobulin A
sIgA: Secretory immunoglobulin A
HGT: horizontal gene transfer
PRRs: pattern recognition receptors
TLR: toll-like receptors
DCs: dendritic cells
TNBS: trinitrobenzene sulfonic acid
Bt: *Bacteroides thetaiotaomicron*
BEVs: *Bacteroides thetaiotaomicron* derived OMVs
UPR: unfolded protein response
ER: endoplasmic reticulum stress
ERAD: endoplasmic reticulum-associated degradation
LPS: lipopolysaccharides
BE: base editor
C: cytosine
U: uracil
PEG: polyethylene glycol

## References

[ref1] Ando H. Lemire S. Pires D. P. Lu T. K. (2015). Engineering modular viral scaffolds for targeted bacterial population editing. Cell Syst 1, 187–196. doi: 10.1016/j.cels.2015.08.013, PMID: 26973885 PMC4785837

[ref2] Anselmo A. C. McHugh K. J. Webster J. Langer R. Jaklenec A. (2016). Layer-by-layer encapsulation of probiotics for delivery to the microbiome. Adv. Mater. 28, 9486–9490. doi: 10.1002/adma.201603270, PMID: 27616140 PMC5287492

[ref3] Barrett K. E. (2010). Building better bugs to deliver biologics in intestinal inflammation. Gut 59, 427–428. doi: 10.1136/gut.2009.195016, PMID: 20332513

[ref4] Bernheim A. Sorek R. (2020). The pan-immune system of bacteria: antiviral defence as a community resource. Nat. Rev. Microbiol. 18, 113–119. doi: 10.1038/s41579-019-0278-2, PMID: 31695182

[ref5] Brown L. Wolf J. M. Prados-Rosales R. Casadevall A. (2015). Through the wall: extracellular vesicles in gram-positive bacteria, mycobacteria and fungi. Nat. Rev. Microbiol. 13, 620–630. doi: 10.1038/nrmicro3480, PMID: 26324094 PMC4860279

[ref1001] Brockstedt D. G. (2004). Listeria-based cancer vaccines that segregate immunogenicity from toxicity. Proc Natl Acad Sci U S A, 101, 13832–7. doi: 10.1073/pnas.0406035101 15365184 PMC518841

[ref6] Cañas M. A. Fábrega M. J. Giménez R. Badia J. Baldomà L. (2018). Outer membrane vesicles from probiotic and commensal *Escherichia coli* activate NOD1-mediated immune responses in intestinal epithelial cells. Front. Microbiol. 9:498. doi: 10.3389/fmicb.2018.00498, PMID: 29616010 PMC5869251

[ref7] Carvalho A. L. Fonseca S. Miquel-Clopés A. Cross K. Kok K. S. Wegmann U.. (2019). Bioengineering commensal bacteria-derived outer membrane vesicles for delivery of biologics to the gastrointestinal and respiratory tract. J Extracell Vesicles 8:1632100. doi: 10.1080/20013078.2019.1632100, PMID: 31275534 PMC6598475

[ref8] Celebioglu H. U. Olesen S. V. Prehn K. Lahtinen S. J. Brix S. Abou Hachem M.. (2017). Mucin- and carbohydrate-stimulated adhesion and subproteome changes of the probiotic bacterium Lactobacillus acidophilus NCFM. J. Proteome 163, 102–110. doi: 10.1016/j.jprot.2017.05.015, PMID: 28533178

[ref9] Ceroni F. Algar R. Stan G. B. Ellis T. (2015). Quantifying cellular capacity identifies gene expression designs with reduced burden. Nat. Methods 12, 415–418. doi: 10.1038/nmeth.3339, PMID: 25849635

[ref10] Chang H. H. Y. Pannunzio N. R. Adachi N. Lieber M. R. (2017). Non-homologous DNA end joining and alternative pathways to double-strand break repair. Nat. Rev. Mol. Cell Biol. 18, 495–506. doi: 10.1038/nrm.2017.48, PMID: 28512351 PMC7062608

[ref11] Charbonneau M. R. Isabella V. M. Li N. Kurtz C. B. (2020). Developing a new class of engineered live bacterial therapeutics to treat human diseases. Nat. Commun. 11:1738. doi: 10.1038/s41467-020-15508-1, PMID: 32269218 PMC7142098

[ref12] Chen D. J. Osterrieder N. Metzger S. M. Buckles E. Doody A. M. DeLisa M. P.. (2010). Delivery of foreign antigens by engineered outer membrane vesicle vaccines. Proc. Natl. Acad. Sci. USA 107, 3099–3104. doi: 10.1073/pnas.0805532107, PMID: 20133740 PMC2840271

[ref13] Chen L. Valentine J. L. Huang C. J. Endicott C. E. Moeller T. D. Rasmussen J. A.. (2016). Outer membrane vesicles displaying engineered glycotopes elicit protective antibodies. Proc. Natl. Acad. Sci. USA 113, E3609–E3618. doi: 10.1073/pnas.1518311113, PMID: 27274048 PMC4932928

[ref14] Chu H. Khosravi A. Kusumawardhani I. P. Kwon A. H. K. Vasconcelos A. C. Cunha L. D.. (2016). Gene-microbiota interactions contribute to the pathogenesis of inflammatory bowel disease. Science 352, 1116–1120. doi: 10.1126/science.aad9948, PMID: 27230380 PMC4996125

[ref15] Claesen J. Fischbach M. A. (2015). Synthetic microbes as drug delivery systems. ACS Synth. Biol. 4, 358–364. doi: 10.1021/sb500258b, PMID: 25079685 PMC4410909

[ref16] Clooney A. G. Sutton T. D. S. Shkoporov A. N. Holohan R. K. Daly K. M. O’Regan O.. (2019). Whole-Virome analysis sheds light on viral dark matter in inflammatory bowel disease. Cell Host Microbe 26, 764–778.e5. doi: 10.1016/j.chom.2019.10.009, PMID: 31757768

[ref17] Conrey P. E. Denu L. O’Boyle K. C. Rozich I. Green J. Maslanka J.. (2023). IgA deficiency destabilizes homeostasis toward intestinal microbes and increases systemic immune dysregulation. Sci Immunol 8:eade2335. doi: 10.1126/sciimmunol.ade2335, PMID: 37235682 PMC11623094

[ref18] Cornuault J. K. Petit M. A. Mariadassou M. Benevides L. Moncaut E. Langella P.. (2018). Phages infecting Faecalibacterium prausnitzii belong to novel viral genera that help to decipher intestinal viromes. Microbiome 6:65. doi: 10.1186/s40168-018-0452-1, PMID: 29615108 PMC5883640

[ref19] Cubillos-Ruiz A. Guo T. Sokolovska A. Miller P. F. Collins J. J. Lu T. K.. (2021). Engineering living therapeutics with synthetic biology. Nat. Rev. Drug Discov. 20, 941–960. doi: 10.1038/s41573-021-00285-3, PMID: 34616030

[ref20] Daeffler K. N. (2017). Engineering bacterial thiosulfate and tetrathionate sensors for detecting gut inflammation. Mol. Syst. Biol. 13:923. doi: 10.15252/msb.20167416, PMID: 28373240 PMC5408782

[ref21] Dedrick R. M. Guerrero-Bustamante C. A. Garlena R. A. Russell D. A. Ford K. Harris K.. (2019). Engineered bacteriophages for treatment of a patient with a disseminated drug-resistant *Mycobacterium abscessus*. Nat. Med. 25, 730–733. doi: 10.1038/s41591-019-0437-z, PMID: 31068712 PMC6557439

[ref22] Díaz-Garrido N. Badia J. Baldomà L. (2021). Microbiota-derived extracellular vesicles in interkingdom communication in the gut. J Extracell Vesicles 10:e12161. doi: 10.1002/jev2.12161, PMID: 34738337 PMC8568775

[ref23] Dion M. B. Oechslin F. Moineau S. (2020). Phage diversity, genomics and phylogeny. Nat. Rev. Microbiol. 18, 125–138. doi: 10.1038/s41579-019-0311-5, PMID: 32015529

[ref24] Doron I. Mesko M. Li X. V. Kusakabe T. Leonardi I. Shaw D. G.. (2021). Mycobiota-induced IgA antibodies regulate fungal commensalism in the gut and are dysregulated in Crohn's disease. Nat. Microbiol. 6, 1493–1504. doi: 10.1038/s41564-021-00983-z, PMID: 34811531 PMC8622360

[ref25] Duraj-Thatte A. M. Courchesne N. D. Praveschotinunt P. Rutledge J. Lee Y. Karp J. M.. (2019). Genetically programmable self-regenerating bacterial hydrogels. Adv. Mater. 31:e1901826. doi: 10.1002/adma.201901826, PMID: 31402514 PMC6773506

[ref26] Durant L. Stentz R. Noble A. Brooks J. Gicheva N. Reddi D.. (2020). Bacteroides thetaiotaomicron-derived outer membrane vesicles promote regulatory dendritic cell responses in health but not in inflammatory bowel disease. Microbiome 8:88. doi: 10.1186/s40168-020-00868-z, PMID: 32513301 PMC7282036

[ref27] Elhenawy W. Debelyy M. O. Feldman M. F. (2014). Preferential packing of acidic glycosidases and proteases into Bacteroides outer membrane vesicles. MBio 5, e00909–e00914. doi: 10.1128/mBio.00909-14, PMID: 24618254 PMC3952158

[ref28] Eskenazi A. Lood C. Wubbolts J. Hites M. Balarjishvili N. Leshkasheli L.. (2022). Combination of pre-adapted bacteriophage therapy and antibiotics for treatment of fracture-related infection due to pandrug-resistant Klebsiella pneumoniae. Nat. Commun. 13:302. doi: 10.1038/s41467-021-27656-z, PMID: 35042848 PMC8766457

[ref29] Esposito G. Pesce M. Seguella L. Lu J. Corpetti C. del Re A.. (2021). Engineered *Lactobacillus paracasei* producing Palmitoylethanolamide (PEA) prevents colitis in mice. Int. J. Mol. Sci. 22. doi: 10.3390/ijms22062945, PMID: 33799405 PMC7999950

[ref30] Federici S. Kredo-Russo S. Valdés-Mas R. Kviatcovsky D. Weinstock E. Matiuhin Y.. (2022). Targeted suppression of human IBD-associated gut microbiota commensals by phage consortia for treatment of intestinal inflammation. Cell 185, 2879–2898.e24. doi: 10.1016/j.cell.2022.07.003, PMID: 35931020

[ref31] Federici S. Kviatcovsky D. Valdés-Mas R. Elinav E. (2023). Microbiome-phage interactions in inflammatory bowel disease. Clin. Microbiol. Infect. 29, 682–688. doi: 10.1016/j.cmi.2022.08.027, PMID: 36191844

[ref32] Forkus B. Ritter S. Vlysidis M. Geldart K. Kaznessis Y. N. (2017). Antimicrobial probiotics reduce *Salmonella enterica* in Turkey gastrointestinal tracts. Sci. Rep. 7:40695. doi: 10.1038/srep40695, PMID: 28094807 PMC5240571

[ref33] Gallagher R. R. Patel J. R. Interiano A. L. Rovner A. J. Isaacs F. J. (2015). Multilayered genetic safeguards limit growth of microorganisms to defined environments. Nucleic Acids Res. 43, 1945–1954. doi: 10.1093/nar/gku1378, PMID: 25567985 PMC4330353

[ref34] Gilmore W. J. Johnston E. L. Bitto N. J. Zavan L. O'Brien-Simpson N. Hill A. F.. (2022). *Bacteroides fragilis* outer membrane vesicles preferentially activate innate immune receptors compared to their parent bacteria. Front. Immunol. 13:970725. doi: 10.3389/fimmu.2022.970725, PMID: 36304461 PMC9592552

[ref35] Gnopo Y. M. D. Watkins H. C. Stevenson T. C. DeLisa M. P. Putnam D. (2017). Designer outer membrane vesicles as immunomodulatory systems - reprogramming bacteria for vaccine delivery. Adv. Drug Deliv. Rev. 114, 132–142. doi: 10.1016/j.addr.2017.05.003, PMID: 28501509

[ref36] Gogokhia L. Buhrke K. Bell R. Hoffman B. Brown D. G. Hanke-Gogokhia C.. (2019). Expansion of bacteriophages is linked to aggravated intestinal inflammation and colitis. Cell Host Microbe 25, 285–299.e8. doi: 10.1016/j.chom.2019.01.008, PMID: 30763538 PMC6885004

[ref37] Guenther C. M. Kuypers B. E. Lam M. T. Robinson T. M. Zhao J. Suh J. (2014). Synthetic virology: engineering viruses for gene delivery. Wiley Interdiscip. Rev. Nanomed. Nanobiotechnol. 6, 548–558. doi: 10.1002/wnan.1287, PMID: 25195922 PMC4227300

[ref38] Guerrero-Mandujano A. Hernández-Cortez C. Ibarra J. A. Castro-Escarpulli G. (2017). The outer membrane vesicles: secretion system type zero. Traffic 18, 425–432. doi: 10.1111/tra.12488 28421662

[ref39] Gunzburg W. H. Aung M. M. Toa P. Ng S. Read E. Tan W. J.. (2020). Efficient protection of microorganisms for delivery to the intestinal tract by cellulose sulphate encapsulation. Microb. Cell Factories 19:216. doi: 10.1186/s12934-020-01465-3, PMID: 33243224 PMC7691082

[ref40] Hanson M. L. Hixon J. A. Li W. Felber B. K. Anver M. R. Stewart C. A.. (2014). Oral delivery of IL-27 recombinant bacteria attenuates immune colitis in mice. Gastroenterology 146, 210–221.e13. doi: 10.1053/j.gastro.2013.09.060, PMID: 24120477 PMC3920828

[ref41] Ho C. L. Tan H. Q. Chua K. J. Kang A. Lim K. H. Ling K. L.. (2018). Engineered commensal microbes for diet-mediated colorectal-cancer chemoprevention. Nat Biomed Eng 2, 27–37. doi: 10.1038/s41551-017-0181-y, PMID: 31015663

[ref42] Hoefler B. C. Stubbendieck R. M. Josyula N. K. Moisan S. M. Schulze E. M. Straight P. D. (2017). A link between Linearmycin biosynthesis and extracellular vesicle genesis connects specialized metabolism and bacterial membrane physiology. Cell Chem Biol 24, 1238–1249.e7. doi: 10.1016/j.chembiol.2017.08.008, PMID: 28919037

[ref43] Hou W. Li J. Cao Z. Lin S. Pan C. Pang Y.. (2021). Decorating Bacteria with a therapeutic Nanocoating for synergistically enhanced biotherapy. Small 17:e2101810. doi: 10.1002/smll.202101810, PMID: 34365713

[ref44] Hsu B. B. Plant I. N. Lyon L. Anastassacos F. M. Way J. C. Silver P. A. (2020). In situ reprogramming of gut bacteria by oral delivery. Nat. Commun. 11:5030. doi: 10.1038/s41467-020-18614-2, PMID: 33024097 PMC7538559

[ref45] Hwang I. Y. Koh E. Wong A. March J. C. Bentley W. E. Lee Y. S.. (2017). Engineered probiotic Escherichia coli can eliminate and prevent Pseudomonas aeruginosa gut infection in animal models. Nat. Commun. 8:15028. doi: 10.1038/ncomms15028, PMID: 28398304 PMC5394271

[ref46] Irene C. Fantappiè L. Caproni E. Zerbini F. Anesi A. Tomasi M.. (2019). Bacterial outer membrane vesicles engineered with lipidated antigens as a platform for Staphylococcus aureus vaccine. Proc. Natl. Acad. Sci. USA 116, 21780–21788. doi: 10.1073/pnas.1905112116, PMID: 31591215 PMC6815149

[ref47] Jiang L. Shen Y. Guo D. Yang D. Liu J. Fei X.. (2016). EpCAM-dependent extracellular vesicles from intestinal epithelial cells maintain intestinal tract immune balance. Nat. Commun. 7:13045. doi: 10.1038/ncomms13045, PMID: 27721471 PMC5062543

[ref48] Jin W. B. Li T. T. Huo D. Qu S. Li X. V. Arifuzzaman M.. (2022). Genetic manipulation of gut microbes enables single-gene interrogation in a complex microbiome. Cell 185, 547–562.e22. doi: 10.1016/j.cell.2021.12.035, PMID: 35051369 PMC8919858

[ref49] Johnston C. D. Cotton S. L. Rittling S. R. Starr J. R. Borisy G. G. Dewhirst F. E.. (2019). Systematic evasion of the restriction-modification barrier in bacteria. Proc. Natl. Acad. Sci. USA 116, 11454–11459. doi: 10.1073/pnas.1820256116, PMID: 31097593 PMC6561282

[ref50] Jones G. R. Bain C. C. Fenton T. M. Kelly A. Brown S. L. Ivens A. C.. (2018). Dynamics of Colon monocyte and macrophage activation during colitis. Front. Immunol. 9:2764. doi: 10.3389/fimmu.2018.02764, PMID: 30542349 PMC6277765

[ref51] Kadurugamuwa J. L. Beveridge T. J. (1996). Bacteriolytic effect of membrane vesicles from *Pseudomonas aeruginosa* on other bacteria including pathogens: conceptually new antibiotics. J. Bacteriol. 178, 2767–2774. 8631663 10.1128/jb.178.10.2767-2774.1996 PMC178010

[ref52] Kaniecki K. De Tullio L. Greene E. C. (2018). A change of view: homologous recombination at single-molecule resolution. Nat. Rev. Genet. 19, 191–207. doi: 10.1038/nrg.2017.92, PMID: 29225334 PMC5866458

[ref53] Kaparakis-Liaskos M. Ferrero R. L. (2015). Immune modulation by bacterial outer membrane vesicles. Nat. Rev. Immunol. 15, 375–387. doi: 10.1038/nri3837, PMID: 25976515

[ref54] Kay M. A. He C. Y. Chen Z. Y. (2010). A robust system for production of minicircle DNA vectors. Nat. Biotechnol. 28, 1287–1289. doi: 10.1038/nbt.1708, PMID: 21102455 PMC4144359

[ref55] Kim H. Kim J. S. (2014). A guide to genome engineering with programmable nucleases. Nat. Rev. Genet. 15, 321–334. doi: 10.1038/nrg3686, PMID: 24690881

[ref56] Kitamoto S. Nagao-Kitamoto H. Jiao Y. Gillilland M. G. III Hayashi A. Imai J.. (2020). The Intermucosal connection between the mouth and gut in commensal Pathobiont-driven colitis. Cell 182, 447–462.e14. doi: 10.1016/j.cell.2020.05.048, PMID: 32758418 PMC7414097

[ref57] Kobayashi H. Kærn M. Araki M. Chung K. Gardner T. S. Cantor C. R.. (2004). Programmable cells: interfacing natural and engineered gene networks. Proc. Natl. Acad. Sci. USA 101, 8414–8419. doi: 10.1073/pnas.0402940101, PMID: 15159530 PMC420408

[ref58] Kong C. Yang M. Yue N. Zhang Y. Tian C. Wei D.. (2024). Restore intestinal barrier integrity: an approach for inflammatory bowel disease therapy. J. Inflamm. Res. 17, 5389–5413. doi: 10.2147/JIR.S470520, PMID: 39161679 PMC11330754

[ref59] Koo T. Yoon A. R. Cho H. Y. Bae S. Yun C. O. Kim J. S. (2017). Selective disruption of an oncogenic mutant allele by CRISPR/Cas9 induces efficient tumor regression. Nucleic Acids Res. 45, 7897–7908. doi: 10.1093/nar/gkx490, PMID: 28575452 PMC5570104

[ref60] Kulp A. Kuehn M. J. (2010). Biological functions and biogenesis of secreted bacterial outer membrane vesicles. Ann. Rev. Microbiol. 64, 163–184. doi: 10.1146/annurev.micro.091208.073413, PMID: 20825345 PMC3525469

[ref61] Kurtz C. B. Millet Y. A. Puurunen M. K. Perreault M. Charbonneau M. R. Isabella V. M.. (2019). An engineered E. coli Nissle improves hyperammonemia and survival in mice and shows dose-dependent exposure in healthy humans. Sci. Transl. Med. 11. doi: 10.1126/scitranslmed.aau7975, PMID: 30651324

[ref62] Lee K. Conboy M. Park H. M. Jiang F. Kim H. J. Dewitt M. A.. (2017). Nanoparticle delivery of Cas9 ribonucleoprotein and donor DNA in vivo induces homology-directed DNA repair. Nat Biomed Eng 1, 889–901. doi: 10.1038/s41551-017-0137-2, PMID: 29805845 PMC5968829

[ref63] Leventhal D. S. Sokolovska A. Li N. Plescia C. Kolodziej S. A. Gallant C. W.. (2020). Immunotherapy with engineered bacteria by targeting the STING pathway for anti-tumor immunity. Nat. Commun. 11:2739. doi: 10.1038/s41467-020-16602-0, PMID: 32483165 PMC7264239

[ref64] Li H. Yang Y. Hong W. Huang M. Wu M. Zhao X. (2020). Applications of genome editing technology in the targeted therapy of human diseases: mechanisms, advances and prospects. Signal Transduct. Target. Ther. 5:1. doi: 10.1038/s41392-019-0089-y, PMID: 32296011 PMC6946647

[ref65] Liao J. Seril D. N. Lu G. G. Zhang M. Toyokuni S. Yang A. L.. (2008). Increased susceptibility of chronic ulcerative colitis-induced carcinoma development in DNA repair enzyme Ogg1 deficient mice. Mol. Carcinog. 47, 638–646. doi: 10.1002/mc.20427, PMID: 18300266 PMC2752943

[ref66] Mager L. F. Burkhard R. Pett N. Cooke N. C. A. Brown K. Ramay H.. (2020). Microbiome-derived inosine modulates response to checkpoint inhibitor immunotherapy. Science 369, 1481–1489. doi: 10.1126/science.abc3421, PMID: 32792462

[ref67] Maggio I. Holkers M. Liu J. Janssen J. M. Chen X. Gonçalves M. A. F. V. (2014). Adenoviral vector delivery of RNA-guided CRISPR/Cas9 nuclease complexes induces targeted mutagenesis in a diverse array of human cells. Sci. Rep. 4:5105. doi: 10.1038/srep05105, PMID: 24870050 PMC4037712

[ref68] Majewska J. Kaźmierczak Z. Lahutta K. Lecion D. Szymczak A. Miernikiewicz P.. (2019). Induction of phage-specific antibodies by two therapeutic staphylococcal bacteriophages administered per os. Front. Immunol. 10:2607. doi: 10.3389/fimmu.2019.02607, PMID: 31803179 PMC6871536

[ref69] Mandell D. J. Lajoie M. J. Mee M. T. Takeuchi R. Kuznetsov G. Norville J. E.. (2015). Biocontainment of genetically modified organisms by synthetic protein design. Nature 518, 55–60. doi: 10.1038/nature14121, PMID: 25607366 PMC4422498

[ref70] Marsh J. W. Ley R. E. (2022). Microbiome engineering: taming the untractable. Cell 185, 416–418. doi: 10.1016/j.cell.2021.12.034, PMID: 35081334

[ref71] Mashburn L. M. Whiteley M. (2005). Membrane vesicles traffic signals and facilitate group activities in a prokaryote. Nature 437, 422–425. doi: 10.1038/nature03925, PMID: 16163359

[ref72] Mazmanian S. K. Round J. L. Kasper D. L. (2008). A microbial symbiosis factor prevents intestinal inflammatory disease. Nature 453, 620–625. doi: 10.1038/nature07008, PMID: 18509436

[ref73] Mills J. Gebhard L. J. Schubotz F. Shevchenko A. Speth D. R. Liao Y.. (2024). Extracellular vesicle formation in Euryarchaeota is driven by a small GTPase. Proc. Natl. Acad. Sci. USA 121:e2311321121. doi: 10.1073/pnas.2311321121, PMID: 38408251 PMC10927574

[ref74] Mimee M. Nagler C. R. (2021). Engineered yeast tune down gut inflammation. Nat. Med. 27, 1150–1151. doi: 10.1038/s41591-021-01420-8, PMID: 34183835

[ref75] Molina-Tijeras J. A. Gálvez J. Rodríguez-Cabezas M. E. (2019). The immunomodulatory properties of extracellular vesicles derived from probiotics: a novel approach for the Management of Gastrointestinal Diseases. Nutrients 11. doi: 10.3390/nu11051038, PMID: 31075872 PMC6567093

[ref76] Motta J. P. (2012). Food-grade bacteria expressing elafin protect against inflammation and restore colon homeostasis. Sci. Transl. Med. 4:158ra144. doi: 10.1126/scitranslmed.3004212 23115353

[ref77] Nandagopal N. Elowitz M. B. (2011). Synthetic biology: integrated gene circuits. Science 333, 1244–1248. doi: 10.1126/science.1207084, PMID: 21885772 PMC4117316

[ref78] Needham B. D. Carroll S. M. Giles D. K. Georgiou G. Whiteley M. Trent M. S. (2013). Modulating the innate immune response by combinatorial engineering of endotoxin. Proc. Natl. Acad. Sci. USA 110, 1464–1469. doi: 10.1073/pnas.1218080110, PMID: 23297218 PMC3557076

[ref79] Nguyen P. Q. Botyanszki Z. Tay P. K. R. Joshi N. S. (2014). Programmable biofilm-based materials from engineered curli nanofibres. Nat. Commun. 5:4945. doi: 10.1038/ncomms5945, PMID: 25229329

[ref80] Nikolich M. P. Filippov A. A. (2020). Bacteriophage therapy: developments and directions. Antibiotics (Basel) 9:3. doi: 10.3390/antibiotics9030135, PMID: 32213955 PMC7148498

[ref81] Nishiyama H. Endo H. Blanc-Mathieu R. Ogata H. (2020). Ecological structuring of temperate bacteriophages in the inflammatory bowel disease-affected gut. Microorganisms 8. doi: 10.3390/microorganisms8111663, PMID: 33121006 PMC7692956

[ref82] Pedrolli D. B. Ribeiro N. V. Squizato P. N. de Jesus V. N. Cozetto D. A. Tuma R. B.. (2019). Engineering microbial living therapeutics: the synthetic biology toolbox. Trends Biotechnol. 37, 100–115. doi: 10.1016/j.tibtech.2018.09.005, PMID: 30318171

[ref83] Pegtel D. M. Gould S. J. (2019). Exosomes. Annu. Rev. Biochem. 88, 487–514. doi: 10.1146/annurev-biochem-013118-111902, PMID: 31220978

[ref84] Piñero-Lambea C. Bodelón G. Fernández-Periáñez R. Cuesta A. M. Álvarez-Vallina L. Fernández L. Á. (2015). Programming controlled adhesion of E. coli to target surfaces, cells, and tumors with synthetic adhesins. ACS Synth. Biol. 4, 463–473. doi: 10.1021/sb500252a, PMID: 25045780 PMC4410913

[ref85] Piraner D. I. Abedi M. H. Moser B. A. Lee-Gosselin A. Shapiro M. G. (2017). Tunable thermal bioswitches for in vivo control of microbial therapeutics. Nat. Chem. Biol. 13, 75–80. doi: 10.1038/nchembio.2233 27842069

[ref86] Pires D. P. Monteiro R. Mil-Homens D. Fialho A. Lu T. K. Azeredo J. (2021). Designing *P. aeruginosa* synthetic phages with reduced genomes. Sci. Rep. 11:2164. doi: 10.1038/s41598-021-81580-2, PMID: 33495501 PMC7835345

[ref87] Riglar D. T. Giessen T. W. Baym M. Kerns S. J. Niederhuber M. J. Bronson R. T.. (2017). Engineered bacteria can function in the mammalian gut long-term as live diagnostics of inflammation. Nat. Biotechnol. 35, 653–658. doi: 10.1038/nbt.3879, PMID: 28553941 PMC5658125

[ref88] Riglar D. T. Silver P. A. (2018). Engineering bacteria for diagnostic and therapeutic applications. Nat. Rev. Microbiol. 16, 214–225. doi: 10.1038/nrmicro.2017.172, PMID: 29398705

[ref89] Rothfield L. Pearlman-Kothencz M. (1969). Synthesis and assembly of bacterial membrane components. A lipopolysaccharide-phospholipid-protein complex excreted by living bacteria. J. Mol. Biol. 44, 477–492. 4899474 10.1016/0022-2836(69)90374-x

[ref90] Saltzman D. A. Heise C. P. Hasz D. E. Zebede M. Kelly S. M. Curtiss R. III. (1996). Attenuated *Salmonella typhimurium* containing interleukin-2 decreases MC-38 hepatic metastases: a novel anti-tumor agent. Cancer Biother. Radiopharm. 11, 145–153. 10851531 10.1089/cbr.1996.11.145

[ref91] Sanna S. van Zuydam N. R. Mahajan A. Kurilshikov A. Vich Vila A. Võsa U.. (2019). Causal relationships among the gut microbiome, short-chain fatty acids and metabolic diseases. Nat. Genet. 51, 600–605. doi: 10.1038/s41588-019-0350-x, PMID: 30778224 PMC6441384

[ref92] Sartorio M. G. Pardue E. J. Feldman M. F. Haurat M. F. (2021). Bacterial outer membrane vesicles: from discovery to applications. Ann. Rev. Microbiol. 75, 609–630. doi: 10.1146/annurev-micro-052821-031444, PMID: 34351789 PMC8500939

[ref93] Scott B. M. Gutiérrez-Vázquez C. Sanmarco L. M. da Silva Pereira J. A. Li Z. Plasencia A.. (2021). Self-tunable engineered yeast probiotics for the treatment of inflammatory bowel disease. Nat. Med. 27, 1212–1222. doi: 10.1038/s41591-021-01390-x, PMID: 34183837

[ref94] Shen Q. Huang Z. Yao J. Jin Y. (2022). Extracellular vesicles-mediated interaction within intestinal microenvironment in inflammatory bowel disease. J. Adv. Res. 37, 221–233. doi: 10.1016/j.jare.2021.07.002, PMID: 35499059 PMC9039646

[ref95] Shen Y. Torchia M. L. G. Lawson G. W. Karp C. L. Ashwell J. D. Mazmanian S. K. (2012). Outer membrane vesicles of a human commensal mediate immune regulation and disease protection. Cell Host Microbe 12, 509–520. doi: 10.1016/j.chom.2012.08.004, PMID: 22999859 PMC3895402

[ref96] Shepherd E. S. DeLoache W. C. Pruss K. M. Whitaker W. R. Sonnenburg J. L. (2018). An exclusive metabolic niche enables strain engraftment in the gut microbiota. Nature 557, 434–438. doi: 10.1038/s41586-018-0092-4, PMID: 29743671 PMC6126907

[ref97] Shkoporov A. N. Clooney A. G. Sutton T. D. S. Ryan F. J. Daly K. M. Nolan J. A.. (2019). The human gut Virome is highly diverse, stable, and individual specific. Cell Host Microbe 26, 527–541.e5. doi: 10.1016/j.chom.2019.09.009, PMID: 31600503

[ref98] Shuwen H. Kefeng D. (2022). Intestinal phages interact with bacteria and are involved in human diseases. Gut Microbes 14:2113717. doi: 10.1080/19490976.2022.2113717, PMID: 36037202 PMC9427043

[ref99] Sinha A. Li Y. Mirzaei M. K. Shamash M. Samadfam R. King I. L.. (2022). Transplantation of bacteriophages from ulcerative colitis patients shifts the gut bacteriome and exacerbates the severity of DSS colitis. Microbiome 10:105. doi: 10.1186/s40168-022-01275-2, PMID: 35799219 PMC9264660

[ref100] Söderblom T. (2005). Effects of the *Escherichia coli* toxin cytolysin a on mucosal immunostimulation via epithelial Ca2+ signalling and toll-like receptor 4. Cell. Microbiol. 7, 779–788. doi: 10.1111/j.1462-5822.2005.00510.x, PMID: 15888081

[ref101] Steidler L. Hans W. Schotte L. Neirynck S. Obermeier F. Falk W.. (2000). Treatment of murine colitis by *Lactococcus lactis* secreting interleukin-10. Science 289, 1352–1355. doi: 10.1126/science.289.5483.1352, PMID: 10958782

[ref102] Steidler L. Robinson K. Chamberlain L. Schofield K. M. Remaut E. le Page R. W. F.. (1998). Mucosal delivery of murine interleukin-2 (IL-2) and IL-6 by recombinant strains of *Lactococcus lactis* coexpressing antigen and cytokine. Infect. Immun. 66, 3183–3189. 9632584 10.1128/iai.66.7.3183-3189.1998 PMC108331

[ref103] Sterner R. C. Sterner R. M. (2021). CAR-T cell therapy: current limitations and potential strategies. Blood Cancer J. 11:69. doi: 10.1038/s41408-021-00459-7, PMID: 33824268 PMC8024391

[ref104] Stirling F. Bitzan L. O’Keefe S. Redfield E. Oliver J. W. K. Way J.. (2017). Rational Design of Evolutionarily Stable Microbial Kill Switches. Mol. Cell 68, 686–697.e3. doi: 10.1016/j.molcel.2017.10.033, PMID: 29149596 PMC5812007

[ref105] Suez J. Zmora N. Zilberman-Schapira G. Mor U. Dori-Bachash M. Bashiardes S.. (2018). Post-antibiotic gut mucosal microbiome reconstitution is impaired by probiotics and improved by autologous FMT. Cell 174, 1406–1423.e16. doi: 10.1016/j.cell.2018.08.047, PMID: 30193113

[ref106] Swirski F. K. Hilgendorf I. Robbins C. S. (2014). From proliferation to proliferation: monocyte lineage comes full circle. Semin. Immunopathol. 36, 137–148. doi: 10.1007/s00281-013-0409-1, PMID: 24435095 PMC3991755

[ref107] Tanna T. Ramachanderan R. Platt R. J. (2021). Engineered bacteria to report gut function: technologies and implementation. Curr. Opin. Microbiol. 59, 24–33. doi: 10.1016/j.mib.2020.07.014, PMID: 32828048

[ref108] Tashiro Y. (2017). Interaction of bacterial membrane vesicles with specific species and their potential for delivery to target cells. Front. Microbiol. 8:571. 28439261 10.3389/fmicb.2017.00571 PMC5383704

[ref109] Thiebes S. Schlesner M. Brors B. Sunyaev A. (2020). Distributed ledger technology in genomics: a call for Europe. Eur. J. Hum. Genet. 28, 139–140. doi: 10.1038/s41431-019-0512-4, PMID: 31527861 PMC6974592

[ref110] Toyofuku M. Nomura N. Eberl L. (2019). Types and origins of bacterial membrane vesicles. Nat. Rev. Microbiol. 17, 13–24. doi: 10.1038/s41579-018-0112-2, PMID: 30397270

[ref111] Toyofuku M. Schild S. Kaparakis-Liaskos M. Eberl L. (2023). Composition and functions of bacterial membrane vesicles. Nat. Rev. Microbiol. 21, 415–430. doi: 10.1038/s41579-023-00875-5 36932221

[ref112] Tran F. Boedicker J. Q. (2017). Genetic cargo and bacterial species set the rate of vesicle-mediated horizontal gene transfer. Sci. Rep. 7:8813. doi: 10.1038/s41598-017-07447-7, PMID: 28821711 PMC5562762

[ref113] Turnbull L. Toyofuku M. Hynen A. L. Kurosawa M. Pessi G. Petty N. K.. (2016). Explosive cell lysis as a mechanism for the biogenesis of bacterial membrane vesicles and biofilms. Nat. Commun. 7:11220. doi: 10.1038/ncomms11220, PMID: 27075392 PMC4834629

[ref114] Van Houdt R. Givskov M. Michiels C. W. (2007). Quorum sensing in Serratia. FEMS Microbiol. Rev. 31, 407–424. doi: 10.1111/j.1574-6976.2007.00071.x, PMID: 17459113

[ref115] Vandenbroucke K. Hans W. van Huysse J. Neirynck S. Demetter P. Remaut E.. (2004). Active delivery of trefoil factors by genetically modified *Lactococcus lactis* prevents and heals acute colitis in mice. Gastroenterology 127, 502–513. doi: 10.1053/j.gastro.2004.05.020, PMID: 15300583

[ref116] Verma P. Greenberg R. A. (2016). Noncanonical views of homology-directed DNA repair. Genes Dev. 30, 1138–1154. doi: 10.1101/gad.280545.116, PMID: 27222516 PMC4888836

[ref117] Wagner J. Maksimovic J. Farries G. Sim W. H. Bishop R. F. Cameron D. J.. (2013). Bacteriophages in gut samples from pediatric Crohn's disease patients: metagenomic analysis using 454 pyrosequencing. Inflamm. Bowel Dis. 19, 1598–1608. doi: 10.1097/MIB.0b013e318292477c, PMID: 23749273

[ref118] Wai S. N. Lindmark B. Söderblom T. Takade A. Westermark M. Oscarsson J.. (2003). Vesicle-mediated export and assembly of pore-forming oligomers of the enterobacterial ClyA cytotoxin. Cell 115, 25–35. doi: 10.1016/S0092-8674(03)00754-2 14532000

[ref119] Waller M. C. Bober J. R. Nair N. U. Beisel C. L. (2017). Toward a genetic tool development pipeline for host-associated bacteria. Curr. Opin. Microbiol. 38, 156–164. doi: 10.1016/j.mib.2017.05.006, PMID: 28624690 PMC5705416

[ref120] Watterlot L. Rochat T. Sokol H. Cherbuy C. Bouloufa I. Lefèvre F.. (2010). Intragastric administration of a superoxide dismutase-producing recombinant *Lactobacillus casei* BL23 strain attenuates DSS colitis in mice. Int. J. Food Microbiol. 144, 35–41. doi: 10.1016/j.ijfoodmicro.2010.03.037, PMID: 20452077

[ref1002] Wallecha A. (2009). Construction and characterization of an attenuated Listeria monocytogenes strain for clinical use in cancer immunotherapy. Clin Vaccine Immunol, 16, 96–103. 19020110 10.1128/CVI.00274-08 PMC2620657

[ref121] Wright R. C. T. Friman V. P. Smith M. C. M. Brockhurst M. A. (2019). Resistance evolution against phage combinations depends on the timing and order of exposure. MBio 10. doi: 10.1128/mBio.01652-19, PMID: 31551330 PMC6759759

[ref122] Wu J. Huang H. Wang L. Gao M. Meng S. Zou S.. (2024). A tailored series of engineered yeasts for the cell-dependent treatment of inflammatory bowel disease by rational butyrate supplementation. Gut Microbes 16:2316575. doi: 10.1080/19490976.2024.2316575, PMID: 38381494 PMC10883098

[ref123] Yehl K. Lemire S. Yang A. C. Ando H. Mimee M. Torres M. D. T.. (2019). Engineering phage host-range and suppressing bacterial resistance through phage tail Fiber mutagenesis. Cell 179, 459–469.e9. doi: 10.1016/j.cell.2019.09.015, PMID: 31585083 PMC6924272

[ref124] Yue H. Jiang J. Taylor A. J. Leite A. D. L. Dodds E. D. du L. (2021). Outer membrane vesicle-mediated Codelivery of the antifungal HSAF metabolites and lytic polysaccharide monooxygenase in the predatory *Lysobacter enzymogenes*. ACS Chem. Biol. 16, 1079–1089. doi: 10.1021/acschembio.1c00260, PMID: 34032403 PMC8797504

[ref125] Zaiss M. M. Joyce Wu H. J. Mauro D. Schett G. Ciccia F. (2021). The gut-joint axis in rheumatoid arthritis. Nat. Rev. Rheumatol. 17, 224–237. doi: 10.1038/s41584-021-00585-3, PMID: 33674813

[ref126] Zhan T. Rindtorff N. Betge J. Ebert M. P. Boutros M. (2019). CRISPR/Cas9 for cancer research and therapy. Semin. Cancer Biol. 55, 106–119. doi: 10.1016/j.semcancer.2018.04.001, PMID: 29673923

[ref1003] Zou L. (2022). PEG-mediated transduction of rAAV as a platform for spatially confined and efficient gene delivery. Biomater Res. 26, 69. doi: 10.1186/s40824-022-00322-1 36461117 PMC9716683

[ref127] Zuo T. Lu X. J. Zhang Y. Cheung C. P. Lam S. Zhang F.. (2019). Gut mucosal virome alterations in ulcerative colitis. Gut 68, 1169–1179. doi: 10.1136/gutjnl-2018-318131, PMID: 30842211 PMC6582748

